# ROS-responsive nanoparticles for oral delivery of luteolin and targeted therapy of ulcerative colitis by regulating pathological microenvironment

**DOI:** 10.1016/j.mtbio.2022.100246

**Published:** 2022-03-23

**Authors:** Chen Tan, Heng Fan, Jiahui Ding, Chaoqun Han, Yang Guan, Feng Zhu, Hui Wu, Yujin Liu, Wei Zhang, Xiaohua Hou, Songwei Tan, Qing Tang

**Affiliations:** a Department of Integrated Chinese and Western Medicine, Union Hospital, Tongji Medical College, Huazhong University of Science and Technology, Wuhan, 430022, China; b Department of Gastroenterology, Union Hospital, Tongji Medical College, Huazhong University of Science and Technology, Wuhan, 430022, China; c School of Pharmacy, Tongji Medical College, Huazhong University of Science and Technology, Wuhan, 430030, China; d Academy of Chinese Medical Sciences, Zhejiang Chinese Medical University, Hangzhou, 310053, China

**Keywords:** ROS-Responsive, Luteolin, Nanoparticle, Ulcerative colitis, Pathological microenvironment

## Abstract

Oxidative stress, caused by excessive production of reactive oxygen species (ROS), plays a crucial role in the occurrence and development of ulcerative colitis (UC). We developed ROS-responsive nanoparticles (NPs) as an efficacious nanomedicine against UC with oral administration. The NPs were fabricated with a d-α-tocopherol polyethylene glycol succinate-b-poly(β-thioester) copolymer (TPGS-PBTE) for ROS cleavage via the colitis-targeted delivery of luteolin (LUT), a natural flavonoid with good anti-inflammation and radical-scavenging activity. Owing to the thioether bond in the polymer main chain, the TPGS-PBTE NPs exhibited an ROS-responsive size change and drug release, which benefited the ROS-scavenging and selective accumulation of LUT in the inflamed colon. In a dextran sulfate sodium-induced acute colitis murine model, LUT@TPGS-PBTE NPs alleviated body weight loss, colon length shortening, and damage to the colonic tissues due to the suppression of ROS and proinflammatory cytokines (e.g., IL-17A, IL-6, interferon-γ, tumor necrosis factor-α), as well as upregulation of glutathione and anti-inflammatory factors (e.g., IL-10, IL-4). More importantly, LUT@TPGS-PBTE NPs regulated the inflammatory microenvironment by modulating the T helper (Th)1/Th2 and Th17/regulatory T cell (Treg) balance (i.e., increased numbers of Tregs and Th2 cells and decreased numbers of Th1 and Th17 ​cells), thus resolving inflammation and accelerating the healing of the intestinal mucosa. Additionally, the LUT@TPGS-PBTE NPs formulation enabled the reduction of the effective dose of LUT and showed excellent biosafety in the mouse model, demonstrating its potential as a targeted UC therapeutic oral preparation.

## Graphical abstract

**Figure:**
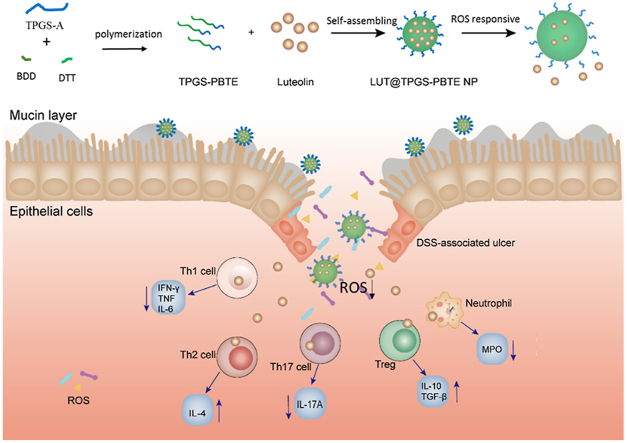

## 1 Introduction

Ulcerative colitis (UC), one of the subtypes of IBD, is a chronic non-specific inflammatory disease of the rectum and colon, affecting the intestinal mucosa and submucosa. Abdominal pain and bloody diarrhea are its major clinical manifestations. The incidence of UC is rising rapidly worldwide, especially in newly industrialized countries [1,2]. Though the pathogenesis of UC is still under research, most researchers believe that the environment, in conjunction with the intestinal flora, acts on genetically susceptible individuals to trigger immune-inflammatory responses and disrupt the gut barrier, leading to the occurrence of UC. Long-term irreversible damage to gastrointestinal structure and function in patients with IBD increases the risk of colon cancer. Current treatments include corticosteroids, aminosalicylic acid (ASA), immunomodulatory drugs, Janus kinase inhibitors, biological agents-monoclonal antibodies against tumor necrosis factor-α (TNF-α), IL-12/23, etc. [3] Biological therapy improves patients’ quality of life and reduces the risk of disease-related complications, including surgery and hospitalization. However, as many as 40% of patients do not respond to the initial treatment. Among the patients whose initial treatment was effective, 13%–46% of them lost their response to the drug in the following year [4]. Therefore, new therapeutic strategies to treat UC are urgently needed.

With the rapid development of nanotechnology, nanoparticles may be promising tools for IBD treatment. Drugs delivered by nanoparticles include miRNA, compounds, biological agents, etc. [5,6] Nanoparticle-based drug delivery systems (DDS) have several significant advantages: (1) They provide high concentrations of local drug in inflamed intestinal regions to prolong pharmacological activity and maximize the efficacy of drugs; (2) targeted drug delivery based on nanocarriers can prevent or reduce the degradation of drugs and loss of their efficacy before reaching the active site; (3) targeted drug delivery in IBD has the potential to reduce the frequency of administration and minimize systemic side effects [5]. Orally administered nano-delivery systems for UC therapy involve different strategies, including pH-dependent, ROS-responsive, Hydrogel-based, active targeting-dependent nano-delivery systems, Saccharide ligands, etc. [5,[7], [8], [9], [10], [11], [12]].

Reactive oxygen species (ROS) have been recognized as a common mechanism in UC [13,14]. Either antioxidants or free radical scavengers are reported as an effective therapeutic agent for UC [15,16]. Moreover, due to the relatively high ROS concentration in UC tissue, ROS responsive system may specifically release drugs in inflamed colon tissues [13]. Literature reported that thioketal nanoparticles (TKNs) could selectively degrade in response to ROS, localize orally delivered siRNA against TNF-α to sites of inflamed colon, and thus inhibit TNF-α expression in inflamed intestinal tissue [17]. Zhang et al. developed a nanotherapy AON (β-cyclodextrin 4-phenylboronic acid pinacol ester based nanoparticle containing a pro-resolving annexin A1-mimetic peptide Ac2-26). It could release packaged Ac2-26 under highly expressed ROS at the lesion sites. This helped to reduce the symptoms of inflammation and accelerate the healing of intestinal mucosal wound [7]. The safety of drug carriers and the drug release kinetics responding to redox stimuli are the major concerns of ROS-responsive drug delivery systems. A drawback of arylboronic esters is the generation of highly reactive intermediates, quinone methide (QM) during degradation. They may react with proteins and DNA and cause some side effects [18]. Sulfur-containing drug carriers are thought to be much safer with a broader ROS responsiveness (arylboronic esters are highly selective to H_2_O_2_) [18,19]. To prepare a system with higher ROS sensitivity than thioketal in order to improve the capability of the targeted drug delivery via oral route, a new thioether containing poly(β-thioester) (PBTE) was synthesized by one-step continuous click chemistry of 1,4-butanediol diacrylate (BDD) and dithiothreitol (DTT) in a mild reaction environment. d-α-tocopheryl polyethylene glycol succinate (TPGS), a biocompatible drug excipient was induced as hydrophilic chain in the form of macromonomer to construct an amphiphilic structure. This can improve the stability of future nanoparticles in water and their tissue permeation [20,21].

Natural active small molecules (NASMs) have shown great potential in UC treatment with advances such as low side effects, low cost and availability [[22], [23], [24]]. However, their instability, lack of targeting and relatively high effective dosage limit their application. Luteolin, a common flavonoid, is widely found in edible plants and Chinese traditional medicine, such as carrots, broccoli, lettuce, honeysuckle, etc. [25,26] Apart from its anti-inflammatory, anti-microbial, anti-viral and anti-tumor properties, luteolin is also a natural antioxidant and has effective radical scavenging and cell protective properties [27]. Structure activity relationship studies indicated that the *ortho*-dihydroxy structure in the B-ring and 2,3-double bond in conjugation with the 4-oxo function of the C-ring makes luteolin to have a good antioxidant capacity [28]. Recent studies have confirmed that LUT can alleviate DSS-induced colitis by inducing ERK1/2 signaling pathway or activating Nrf2 signaling pathway, as well as regulating the composition of intestinal flora to reduce inflammation, apoptosis and autophagy [26,29,30]. Moreover, LUT has the ability to regulate CD4^+^ T cell subsets in some cases, including acute lung injury, allograft rejection, allergic asthma, etc. [[31], [32], [33]] Unfortunately, like most flavonoids, LUT is prone to oxidation and degradation due to its highly unsaturated structure, and it is limited by its poor water solubility, low bioavailability and short half-life. UC can only be attenuated with a high dosage of LUT (50 ​mg/kg) via oral administration; while 20 ​mg/kg LUT has little effect on it [26]. How to prepare a new oral preparation and then effectively deliver LUT to UC lesion is of great value in practicing how to remodel the balance of CD4^+^ T lymphocytes. This may provide a new approach to UC nonatherapy more than resolving inflammation as done by previously reported oral nanoparticles [7,8,17].

**Scheme 1:**
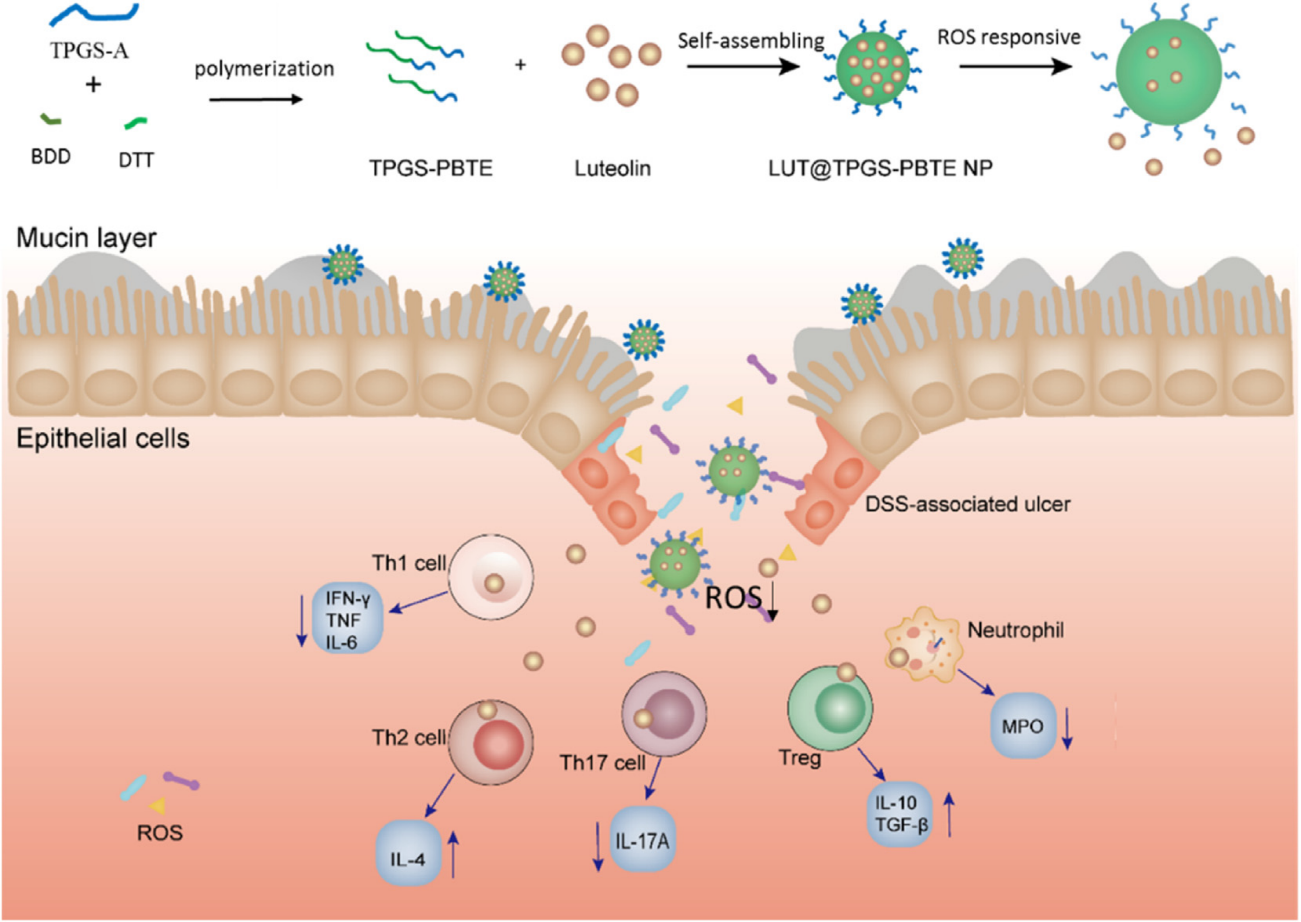
Preparation of LUT@TPGS-PBTE NPs and schematic illustration of UC treatment mechanism.

Here, we first synthesized a ROS-responsive thioether containing copolymer, TPGS-PBTE, with a new main chain structure. Then, LUT was loaded to form LUT@TPGS-PBTE NPs and the possibility of using thioether based nanomedicine for UC treatment via oral administration was evaluated for the first time. Such system could treat UC by cleaving ROS, releasing LUT, suppressing inflammation and modulating the immune microenvironment (Scheme 1). The particle size, zeta-potential, stability, ROS responsiveness and biodistribution of TPGS-PBTE NPs were assessed. The anti-UC efficacy of LUT@TPGS-PBTE NPs was evaluated in dextran sulfate sodium (DSS)-induced acute colitis murine model. The molecular mechanism and modulation of the pathological microenvironment of this system used for UC treatment were also explored.

## 2 Materials and methods

### 2.1 Materials

Luteolin and DTT were obtained from Aladdin (China). 1,4-butanediol diacrylate (BDD) was purchased from TCI (Shanghai, China). Dextran sulfate sodium (DSS, 35000 ​Da) was purchased from MP Biomedical (USA). Mouse Th1/Th2/Th17 CBA Kit, Cytofix/CytopermSoln Kit, Leuko Act Cktl with GolgiPlug, Transcription Factor Buffer Set, Fixable Viability Stain 780, FITC-Anti-CD4 antibody, BV605-Anti-IL-17A antibody, APC-Anti–INF–γ antibody and PE-Anti-IL-4 antibody were purchased from BD Biosciences (San Diego, USA). PE-Anti-FOXP3 antibody was purchased from eBiosciences (San Diego, CA). Myeloperoxidase (MPO) kit, glutathione (GSH) kit and ROS kit were supplied by Nanjing Jiancheng Bioengineering Institute (Nanjing, China). The BCA protein determination kit and Reactive Oxygen Species Assay Kit were purchased from Beyotime Biotechnology (China). Mouse Beta Actin antibody, Mouse Occludin antibody and Mouse ZO-1 antibody for western blotting were purchased from Proteintech (China). SYBR Premix Ex Taq™, Trizol reagent and PrimeScript™ RT 131 Master Mix were obtained from TaKaRa Bio (China). All other reagents are commercially available and can be used directly. All the solvents used were of analytical grade and were procured from Sinopharm (China).

### 2.2 Synthesis and characterization of TPGS-PBTE

TPGS acrylate (TPGS-A) was first synthesized by TPGS and acryloyl chloride. Then, TPGS-A (158 ​mg, 0.1 ​mM) was applied as a macromonomer and co-dissolved with 396 ​mg BDD (2 ​mM) and 317 ​mg DTT (2.05 ​mM) in 3 ​mL ​N, *N*-dimethylformamide (DMF). After adding trace hexylamine and reacting for 4 ​h at room temperature, the solution was transferred to a dialysis tube (MWCO 2000). It was dialyzed in water/DMF mixture (50:50, v: v) twice and in water thrice. The outer phase was changed every 3 ​h. The final product, TPGS-PBTE was collected by lyophilization.

To synthesize PBTE homopolymer, 198 ​mg BDD (1 ​mM) and 154 ​mg DTT (1 ​mM) were reacted just like TPGS-PBTE. The chemical structures of the products were characterized by 1H NMR (Bruker AVANCE III 400 ​MHz NMR spectrometer, solvents: CDCl3). The molecular weight of TPGS-PBTE was measured by gel permeation chromatography (GPC, Waters-2410 system) with a Waters 2414 refractive index detector (mobile phase: DMF, standard: narrow-disperse polystyrene). Non-ROS responsive copolymer, TPGS-PLGA, was synthesized by conjugating TPGS and carboxyl ended PLGA using DCC and DMAP as catalyst.

### 2.3 Preparation, characterization and drug loading of TPGS-PBTE NPs

Luteolin-loaded TPGS-PBTE nanoparticles (LUT@TPGS-PBTE NPs) were prepared by a modified nanoprecipitation method. In brief, 3 ​mg luteolin and 10 ​mg TPGS-PBTE were dissolved in 400 ​μl DMF. The obtained solution was added dropwise to 5 ​mL HCl solution (pH ​= ​4.0) containing 0.03% TPGS (w/v). The suspension was stirred at room temperature for 10 ​min, and then dialyzed (MWCO 1000) against 500 ​ml of deionized water for 15 ​min. This was repeated 3 times. Aggregations were removed by centrifugation at 500 ​rpm for 3 ​min, and the supernatant was centrifuged at 12000 ​rpm for 10 ​min. The obtained precipitate was redispersed in PBS and repeated 3 times to remove free LUT. Following similar procedures, DiR-labeled TPGS-PBTE NPs (DiR@TPGS-PBTE NPs), coumarin-6-labeled TPGS-PBTE NPs (COU@TPGS-PBTE NPs) as well as different TPGS-PLGA NPs loaded with LUT, DiR or coumarin-6 were separately prepared.

Particle size and ζ-potential of the formulations were characterized by dynamic light scattering (DLS, Zeta PALs, Brookhaven, USA). The morphologies of LUT@TPGS-PBTE NPs and TPGS-PBTE NPs were observed by transmission electron microscope (TEM, JEM-1230, Japan).

The quantitative analysis of LUT was performed using a fluorescence spectrophotometer by UV detection at 350 ​nm. Before test, 500 ​μL LUT@TPGS-PBTE NPs were dissolved in 4.5 ​ml DMF. The concentrations of the drugs were calculated according to their standard curves. Lyophilized NPs were dissolved in DMF before analysis. The drug loading amount (DL%) was defined as follows:

To investigate the ROS responsive property of the nanoparticles, TPGS-PBTE NPs and LUT@TPGS-PBTE NPs were incubated with/without 1 ​mM ​H_2_O_2_. Then, their particle size was monitored by DLS and their morphology was observed by TEM. To further reveal the chemical change of PBTE, PBTE NPs were prepared like TPGS-PBTE NPs. Then, they were incubated with/without 1 ​mM ​H_2_O_2_ for 24 ​h. The PBTE NPs solution was lyophilized and their powder was analyzed by 1H NMR (solvents: DMSO-*d*_6_) and mass spectrometry (MS, Advion Expression L, China).

### 2.4 *In vitro* release of LUT

After the amount of LUT was determined, 1 ​ml of fresh LUT@TPGS-PBTE NPs and LUT@TPGS-PLGA NPs solution was placed in a dialysis bag (MWCO 3500) and dialyzed against 50 ​mL of glycine-HCl buffer (pH 2.0) for the first 2 ​h. It was at first transferred to PBS (pH 6.8) for 2 ​h, and finally to PBS (pH 7.4) for 18 ​h with/without 1 ​mM H_2_O_2_. Within each time interval, 3 ​ml of external phase was taken out to detect the concentration of LUT and 3 ​ml of fresh buffer was simultaneously supplied. The release rate of LUT was then calculated and the *in vitro* release curve was drawn.

### 2.5 Cell culture and stimulation

The Caco-2 ​cells were purchased from the American Type Culture Collection (ATCC) and maintained in Dulbecco's Modified Eagle Medium (DMEM) (Gibco BRL, Life Technologies, USA) containing 10% fetal bovine serum (Gibco® Cell Culture, Melbourne, VIC, Australia) with 1% penicillin and streptomycin (Gibco, Carlsbad, CA, USA). They were cultured at 37 ​°C in a 5% CO2 atmosphere. Murine TNF-α (100 ​ng/ml, Peprotech) was incubated with Caco-2 ​cells for 24 ​h to build inflammatory cell model.

### 2.6 Cellular uptake of fluorescent nanoparticles

For qualitative investigation of the cellular uptake efficiency of coumarin-6-loaded NPs, Caco-2 ​cells and their inflammatory model were incubated with free coumarin-6, coumarin-6-loaded TPGS-PBTE NPs (COU@TPGS-PBTE NPs) or coumarin-6-loaded TPGS-PLGA NPs (COU@TPGS-PLGA NPs) with the coumarin-6 dosage of 300 ​ng/ml at 37 ​°C for 1,2,4 ​h. Then, they were washed with PBS thrice. Nuclei were stained with DAPI. Confocal laser scanning microscope (Olympus FV1000) was used to visualize the fluorescence.

For flow cytometric (FCM) assay, Caco-2 ​cells were seeded in six-well plates and incubated as mentioned above. Then, the cells were collected and washed with PBS, and the intracellular fluorescence of coumarin-6 was detected by flow cytometer (BD PharMingen, San Diego, CA, United States). The fluorescence intensity of coumarin-6 was collected at 488 ​nm excitation and 575 ​nm bandpass filter. Two thousand viable events were collected and analyzed using FlowJo V10 software (Tree Star, Ashland, OR, United States).

### 2.7 Western blotting assay

The cellular tight junction proteins (occluding and ZO-1) in Caco-2 ​cells were detected by western blotting as previously described [34]. In brief, proteins were collected from Caco-2 ​cells with RIPA Lysis Buffer (Beyotime, China) containing 1%(v/v) phenylmethyl sulfonyl fluoride (PMSF). Protein concentrations were detected using a the bicinchoninic acid (BCA) assay kit (Beyotime, China). Equal amounts of denatured protein samples were separated by sodium dodecyl sulfate polyacrylamide gel electrophoresis (SDS-PAGE) and then electrophoretically transferred onto PVDF membranes (Millipore Corp., MA, USA). After blocking with 5% nonfat milk in TBS buffer containing 0.1% Tween-20, the membrane was immunodetected with rabbit anti-Occludin antibody (1:1000, Proteintech) and rabbit anti-ZO-1 antibody (1:1000, Proteintech), and mouse anti-β-actin antibody (1:1000, Proteintech) was for normalization. Afterwards, the membranes were incubated with goat anti-rabbit secondary antibodies (1:3000, Proteintech) and goat anti-mouse secondary antibodies (1:3000, Proteintech). Protein bands were visualized by enhanced chemiluminescence (ECL) kit (Beyotime, China) and subjected to an Image Reader LAS-4000 imaging system (FUJIFLIM, Japan).

### 2.8 Animals

Male Kunming mice weighing 22–25 ​g (7–8 weeks old) were purchased from Hubei Provincial Center for Disease Control and Prevention. The animals were kept at the laboratory animal center of Huazhong University of Science and Technology (Huazhong University of science and technology, Wuhan). The animals were housed in standard mouse cages under conditions of optimum light (12:12 ​h light-dark cycle), temperature (24–25 ​°C), and humidity (70–75%), with *ad libitum* access to water and food. In this study, all procedures involving animals and their care were strictly guided by the HUST Institute of Zoology Board Guidelines and approved by the HUST Institutional Animal Care and Use Committee (IACUC Number: 2527). Before further experiments, all mice were acclimatized for at least 7 days. All surgical procedures were performed under anesthesia to minimize pain. Ulcerative colitis was induced in the mice by giving them drinking water containing 3% (w/v) DSS for 7 days, according to the method published by Wirtz [35].

### 2.9 *In vivo* localization and biodistribution of nanoparticles

*In vivo* localization and bio-distribution of the nanoparticles was investigated in acute UC model mice and healthy mice. Briefly, acute colitis was induced in the mice as stated above. After 7 days of DSS processing, the mice received orally administered Dir@TPGS-PBTE NPs, Dir@TPGS-PLGA NPs or free Dir, respectively. Then, their blood, hearts, livers, spleens, lungs, kidneys and colons were harvested at predetermined time intervals (0, 1, 2, 4, 8, 12 ​h). All the organs were washed in PBS to remove blood and feces. Each mouse was given the same dosage of Dir (2 ​mg/kg) and the results were analyzed by IVIS software (IVIS, Caliper, USA).

The localization of orally delivered NPs in the gastrointestinal tract of both healthy and UC mice was further observed by fluorescence microscope (Olympus, Tokyo, Japan) to evaluate the selective accumulation of free Dir, Dir@TPGS-PBTE NPs and Dir@TPGS-PLGA NPs in the inflamed colon. Prior to the administration, the mice fasted for 12 ​h. After 12 ​h of administration, the mice were euthanized and segments of their distal colon were isolated. Tissues were then embedded in Tissue-Tek® O·C.T. compound and frozen at −80 ​°C for subsequent experiments. Cryosections were cut at a thickness of 5 ​μm using a cryostat microtome and nuclei were stained with DAPI; the images were taken on a fluorescence microscope.

### 2.10 UC treatment and assessment of inflammation

The animals were randomly assigned into six groups: control group (Control), DSS induced colitis group (Colitis), free luteolin treated group (LUT), LUT and TPGS-PBTE NPs mixture treated group (LUT ​+ ​TPGS-PBTE NPs), as well as LUT@TPGS-PBTE NPs and LUT@TPGS-PLGA NPs treated groups. The groups were orally given saline, free LUT, LUT ​+ ​TPGS-PBTE NPs, LTU@TPGS-PBTE NPs and LUT@TPGS-PLGA NPs for 7 days, respectively. All the formulations were resuspended in PBS and the dosage of LUT is 20 ​mg/kg.

During the treatments, changes in the body weight, stool viscosity, and hematochezia status of the mice were monitored daily to score the disease activity index. Disease activity index (DAI) is defined as the summation of the stool consistency index (0–3), fecal bleeding index (0–3), and weight loss index (0–4) [35]. On day 15, the mice were euthanized, and the entire colons (from the cecum to the rectum) were collected. Mesenteric lymph nodes (MLNs) and spleens of the mice were collected for flow cytometry analysis. Parts of the colons were fixed with 4% paraformaldehyde, stained with hematoxylin and eosin (H&E) and then observed by optical microscopy. Histological analyses were performed as described previously. The rest of the colon tissues were frozen in liquid nitrogen and stored at −80 ​°C for subsequent analysis. Besides, the heart, liver, spleen, lungs and kidneys of each mouse were collected and stained with H&E for histopathological analysis. Whole blood and serum were collected for blood routine and biochemical tests.

MPO activity was measured using the MPO kit to quantify colonic neutrophil infiltration according to the manufacturer's instructions. The ROS formed in the colon tissues and Caco-2 ​cells were obtained with dichlorofluorescein diacetate (DCF-DA) at an excitation wavelength of 485 ​nm and emission wavelength of 525 ​nm, respectively. Glutathione content was recorded at 405 ​nm by a microplate reader.

Immunofluorescence staining was conducted as previously described [36]. Briefly, 4% paraformaldehyde was used to fix fresh colon tissue samples from the mice. Paraffin-embedded sections (5 ​μm) were dewaxed and rehydrated through graded alcohols. Antigen heat recovery was conducted in citrate buffer with pressure cooker and then cooled down to room temperature. Endogenous antigen was blocked with 10% donkey serum for 1 ​h. Occludin (1:200, A2601, ABclonal), ZO-1(1:200, AF5145, Affinity) and claudin-1 (1:200,13050-1-AP, PTG) were used as primary antibodies, respectively. Confocal laser scanning microscope (Olympus FV1000) was applied to examine the expression levels of the proteins.

The levels of mRNA of IL-10, TNF-α, TGF-β, IL-4, Foxp3, IL-1β, IL-17A and IFN-γ in the colonic tissues were measured by real-time polymerase chain reaction (qRT-PCR). β-actin was used as an internal control. Briefly, total RNA was isolated from the colonic tissues with TRIzol (TaKaRa, Dalian, China). Using PrimeScript ™ RT Master Mix (Takara), it was reverse transcribed to obtain complementary DNA (cDNA). Subsequently, cDNA fragments were amplified with SYBR Premix Ex TaqTM (TaKaRa)and analyzed to determine gene expression changes. The Caco-2 ​cells and their inflammatory model were treated *in vitro* with LUT, LUT ​+ ​TPGS-PBTE NPs, LUT@TPGS-PBTE NPs and LUT@TPGS-PLGA NPs respectively, and the mRNA levels of NF-κB, TNF-α, IL-1β and IL-6 were detected as previously described. The sequences of the primers are listed in Table S1.

### 2.11 Analysis of Th1/Th2/Th17/Treg by CBA and flow cytometry

To determine the concentration of the cytokines including IL-2, IFN-γ, IL-4, IL-6, TNF, IL-17A and IL-10 in the colons and serum of the animals, the BD™ Cytometric Bead Array (CBA) Mouse Th1/Th2/Th17 Cytokine Kit was used according to the manufacturer's instruction. Data were analyzed with FCAP Array software.

For flow cytometry assay, mesenteric lymph nodes (MLNs) and spleen single-cell suspensions were harvested by gently pushing cells through a 70-μm cell strainer with a syringe piston as previously described [34]. For Treg analysis, cells were stained with FITC conjugated anti-CD4 antibody at 4 ​°C in the dark for 20 ​min. They were fixed and permeabilized for 40 ​min in the dark, and finally stained with PE conjugated anti-FOXP3 antibody. For Th1/Th2/Th17 analysis, cells were first stimulated with a leukocyte activation cocktail in 5% CO_2_ at 37 ​°C for 6 ​h. Subsequently, they were stained with FITC conjugated anti-CD4 antibody (BD Biosciences) for 30 ​min in the dark, and then fixed and permeabilized for 40 ​min. Finally, they were stained with APC conjugated anti–IFN–γ antibody (BD Biosciences), BV605 conjugated anti-IL-17A antibody (BD Biosciences), and PE conjugated anti-IL-4 antibody (BD Biosciences). After washing with permeabilization buffer (BD Biosciences), the stained cells were analyzed by flow cytometer.

### 2.12 Statistical analysis

All data were reported as mean ​± ​standard deviation (Mean ​± ​SD). Two groups were analyzed with unpaired two tailed Student t-test. The data sets including more than two groups of data were analyzed by one-way ANOVA and Bonferroni post hoc test. P ​< ​0.05 was considered statistically significant. SPSS 22.0 was used for data analysis.

## 3 Results

### 3.1 Fabrication and characterization of TPGS-PBTE NPs

**Fig. 1:**
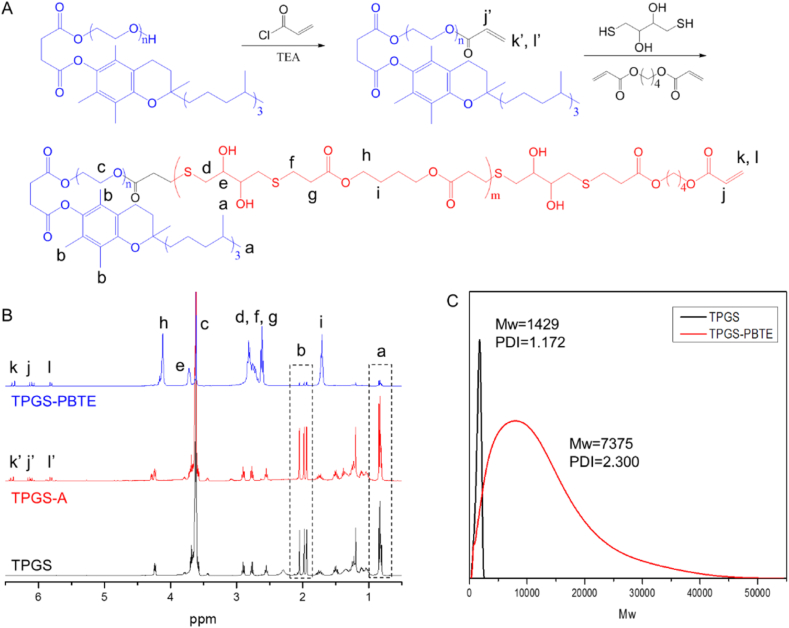
Synthesis and characterization of TPGS-PBTE. A) Synthetic scheme of TPGS-PBTE. B)1H NMR spectra of TPGS, TPGS-A and TPGS-PBTE. C) GPC curves of TPGS and TPGS-PBTE.

Macromonomer TPGS-A was first synthesized as described above. As shown in Fig. 1A, new peaks between 5.80 and 6.50 ​ppm due to the –CH=CH_2_ group of TPGS acrylate occurred compared to TPGS. This confirmed the formation of TPGS-A. TPGS-PBTE was then synthesized through Michael addition polymerization using BDD, DTT and TPGS-A (Fig. 1A) at acrylate-thiolmolar ratio of 1:1 to obtain the highest molecular weights [37]. Due to the fast reaction kinetics led by the click chemistry between acrylate and thiol group, the polymerization proceeded at room temperature and was finished in hours [38]. In the 1H NMR spectrum (Fig. 1B) of TPGS-PBTE, the typical peaks of TPGS (0.86, 1.90–2.10 and 3.61 ​ppm), BDD (4.11 ​ppm, –OCOCH_2_−) and DTT (3.71 ​ppm, –CH_2_CH(OH) CH−) units were all found, which confirmed the successful synthesis of TPGS-PBTE. It can be also seen from GPC (Fig. 1C) that the average molecular weight (Mw) of TPGS-PBTE was 7375, while the Mw of TPGS was 1429. This preliminarily proved that small molecular monomers were polymerized on TPGS.

### 3.2 Characterization and ROS-sensitivity of TPGS-PBTE NPs

**Fig. 2:**
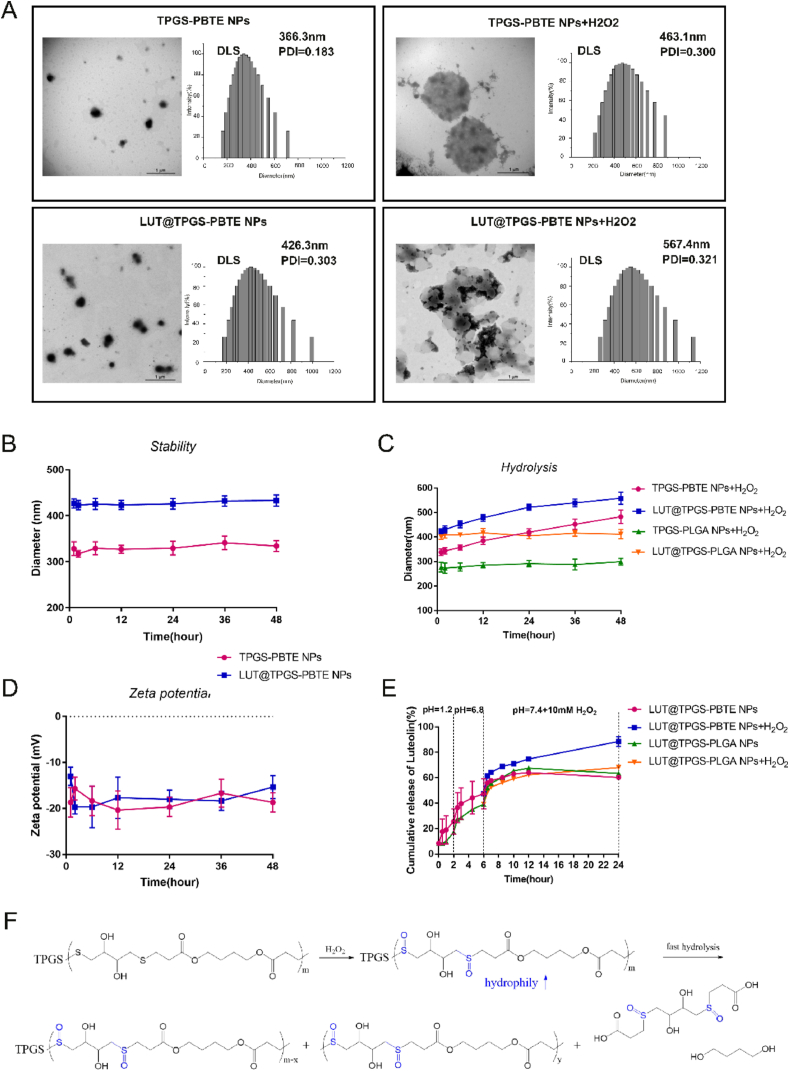
Characterization and ROS-sensitivity of TPGS-PBTE NPs and *in vitro* drug release of LUT@TPGS-PBTE NPs. A) TEM images and DLS results of TPGS-PBTE NPs and LUT@TPGS-PBTE NPs. B) Stability of TPGS-PBTE NPs and LUT@TPGS-PBTE NPs in PBS during 48 ​h. C) Hydrolysis of NPs in the presence of 1 ​mM hydrogen peroxide. D)Zeta potential of TPGS-PBTE NPs and LUT@TPGS-PBTE NPs in PBS during 48 ​h. E) In vitro LUT release of LUT@TPGS-PBTE NPs and LUT@TPGS-PLGA NPs in buffers simulating the gastrointestinal pH conditions with/without 1 ​mM hydrogen peroxide. F) Schematic diagram of hydrolysis of TPGS-PBTE NPs. Data were expressed as mean ​± ​SD (n ​= ​3).

LUT@TPGS-PBTE NPs and TPGS-PBTE NPs were prepared by a modified nano-precipitation method and the drug loading amount (DL%) of LUT was 13.4%. Probably due to the polyphenol structure of LUT, whose solubility decreased under weak acid condition, LUT loading amount reach its maximum in HCl solution (pH 4.0), which was much higher than in double distilled water (ddH_2_O) and PBS (Figure S1). As shown in Fig. 2A, both TPGS-PBTE NPs and LUT@TPGS-PBTE NPs have a spherical structure with a diameter of about 230 and 300 ​nm. These are smaller than those of DLS results (360 ​nm and 430 ​nm), probably caused by the dehydration and shrink of NPs. In addition, TPGS-PBTE NPs and LUT@TPGS-PBTE NPs displayed negative zeta potential (Fig. 2D), which may be caused by the TPGS out-layer. Storage stability test of LUT@TPGS-PBTE NPs and TPGS-PBTE NPs revealed that the diameter of the two remained unchanged for 48 ​h, indicating the nanoparticles had high stable structures (Fig. 2B).

Then we evaluated *in vitro* ROS-responsive hydrolysis characteristics of TPGS-PBTE NPs and LUT@TPGS-PBTE NPs (Fig. 2C). The change in the particle size of the NPs with the addition of 1 ​mM H_2_O_2_ buffer was continuously detected at different incubation times. Different from the stability test, where almost no changes were observed in TPGS-PBTE NPs and LUT@TPGS-PBTE NPs, the particle size of TPGS-PBTE NPs and LUT@TPGS-PBTE NPs obviously enlarged in the presence of H_2_O_2_. Although the average diameter measured by DLS only increased by 100 ​nm, the detailed result of TEM proved the formation of aggregations (∼1 ​μm). This could be due to the oxidation of the thioether bond by H_2_O_2_ and formation of sulfone bond, which increased the hydrophilicity of NPs, accelerated the hydrolysis of ester bond and thus decreased the molecular weight of the copolymer [38]. As a result, the hydrophilic-lipophilic balance of TPGS-PBTE NPs system was disrupted and presented as the particle size changed.

To further reveal the ROS responsive mechanism, PBTE was treated with H_2_O_2_ and investigated by ^1^H NMR and MS. As shown in Figure S2, after incubating PBTE in water for 24 ​h, no change was observed in its NMR spectrum. However, the characteristic peak of DTT unit in H_2_O_2_ treated PBTE shifted to lower field. Due to the intramolecular/intermolecular hydrogen bond between –OH and S=O, the proton signal of –OH increased from 4.74 ​ppm to 5.24/5.35 ​ppm. In addition, the proton signals of –CH2- next to sulfur (a, d) and proton in tertiary carbon (b) of DTT unit all shifted to lower field, indicating the oxidation of the thioether bond. We also utilized MS to confirm the chemical structure of PBTE after H_2_O_2_ treatment (Figure S3). The fragment ion peak at 216 and 168 indicated the formation of sulfone in PBTE backbone. Molecular ion peak at 330 and fragment ion peak at 285 and 257 confirmed the existence of 3,3'-(2,3-dihydroxybutane-1,4-diyldisulfinyl) dipropionic acid, the typical hydrolysis product of the oxidized PBTE.

In addition, there were no significant changes in the diameter of TPGS-PLGA NPs and LUT@TPGS-PLGA NPs in the presence of hydrogen peroxide. All these results demonstrated the ROS responsiveness of TPGS-PBTE, which benefited its application in drug delivery targeting colitis.

### 3.3 *In vitro* drug release of LUT@TPGS-PBTE NPs

The *in vitro* release of luteolin from LUT@TPGS-PBTE NPs and LUT@TPGS-PLGA NPs is presented in Fig. 2E. To simulate the pH of the gastrointestinal tract, NPs was successively placed in buffers with different pH values (pH2.0, 6.8, 7.4) on a shaker at 37 ​°C. At each time point, the released LUT was measured using an ultraviolet spectrophotometer at 350 ​nm. The drug release curves of all the NPs showed sustained release behavior. In the first 2 ​h at pH 2.0 (simulating stomach), the release amount of LUT from LUT@TPGS-PBTE NPs and LUT@TPGS-PLGA NPs was 25.5 ​± ​9.9% and 17.1 ​± ​0.34%, respectively. After 2–6 ​h (pH 6.8, simulating intestine) of incubation, the cumulative release amount was 47.45 ​± ​11.8% and 39.1 ​± ​0.64% from LUT@TPGS-PBTE NPs and LUT@TPGS-PLGA NPs, showing no significant difference. When switching to PBS (pH7.4, simulating colon, without H_2_O_2_), the cumulative release amount of LUT from LUT@TPGS-PBTE NPs and LUT@TPGS-PLGA NPs was 60.3 ​± ​0.6% and 63.5 ​± ​0.9%, respectively. However, LUT was significantly released in the presence of H_2_O_2_ (simulating inflammatory colon); 88.6 ​± ​3.9% loaded LUT was released, which was 1.5 times higher than that without H_2_O_2_. On the other hand, even with 1 ​mM H_2_O_2_, LUT@TPGS-PLGA NPs only released 68.1 ​± ​1.0% of LUT, which was almost the same as that released without H_2_O_2_. This is in line with the results in the last section that TPGS-PLGA showed no ROS responsiveness. These results demonstrated that LUT released from LUT@TPGS-PBTE NPs was highly responsive to peroxide, indicating the capacity of TPGS-PBTE NPs to deliver the loaded drug to the inflamed tissues with oxidative stress.

### 3.4 Accumulation of TPGS-PBTE NPs in the inflamed colon

**Fig. 3:**
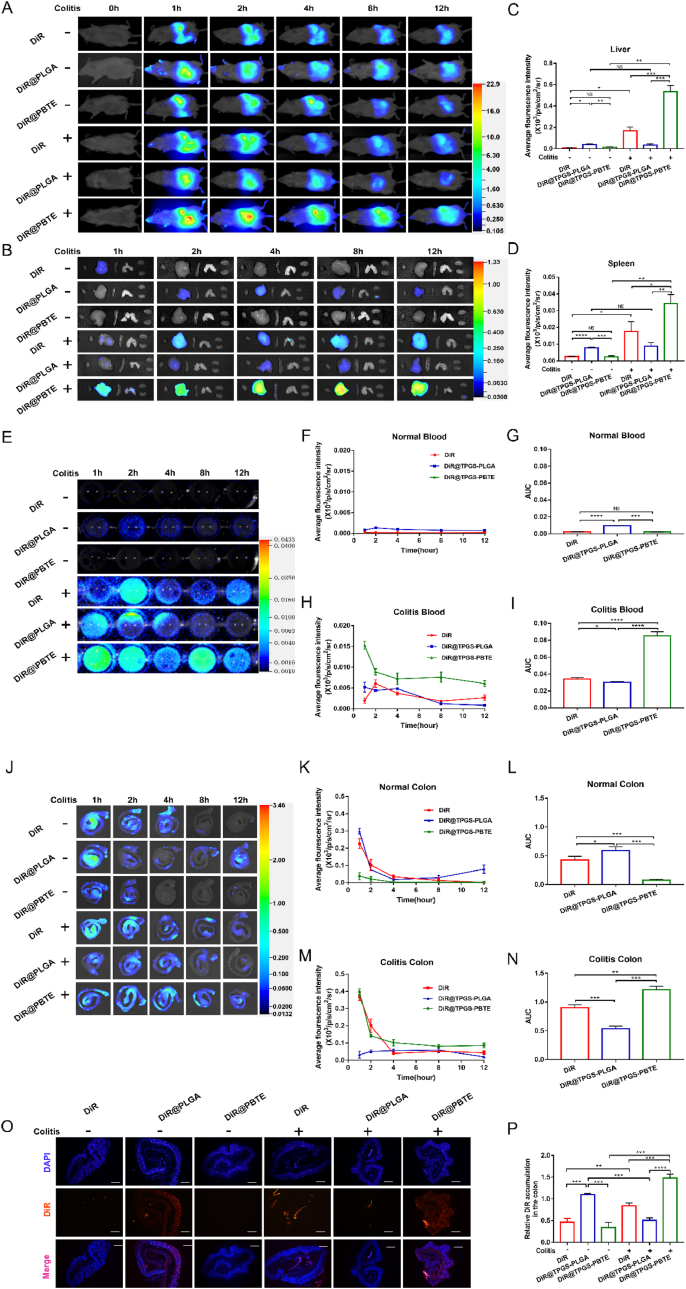
Selective accumulation of TPGS-PBTE NPs in the inflamed colon. A) In vivo imaging of healthy and colitis mice after gavage administration of free DiR, DiR@TPGS-PBTE NPs and DiR@TPGS-PLGA NPs. B) Representative ex vivo images of different organs (from left to right: heart, liver, spleen, lungs, kidneys).C-D) Average fluorescence intensity in liver and spleen at 12 ​h after gavage. E) Fluorescence Images of peripheral blood from healthy and colitis mice. F–I) Quantitative analysis of fluorescence intensity in healthy blood (F and G) and colitis blood (H and I). J) Representative fluorescence Images of colon from healthy and colitis mice. K–N) Quantitative analysis of fluorescence intensity in healthy colon (K and L) and colitis colon (M and N). O) Fluorescence images of cryosections of colonic tissues from healthy and colitis mice 12 ​h after treatments (Bar ​= ​200 ​μm). P) Relative DiR intensity in colonic tissues after 12 ​h of oral administration in healthy and colitis mice. Data were expressed as mean ​± ​SD; ∗p ​< ​0.05, ∗∗p ​< ​0.01, ∗∗∗p ​< ​0.001, ∗∗∗∗p ​< ​0.0001; NS, no significance.

To evaluate the selective accumulation of TPGS-PBTE NPs in the inflamed colon tissues, in vivo and ex vivo imaging was performed using Dir as fluorescence probe. Initially, both healthy and colitis mice were given free DiR, DiR-labeled TPGS-PBTE NPs (DiR@TPGS-PBTE NPs) and DiR-labeled TPGS-PLGA NPs (DiR@TPGS-PLGA NPs) by gavage, respectively. As shown in Fig. 3A, during 12 ​h after oral administration, similar distribution tendency was observed in free Dir and DiR@TPGS-PBTE NPs groups in both healthy and colitis mice. Fluorescence started to accumulate in the lower abdomen of UC models within 1 ​h, and the strength became weak at about 4 ​h. In contrast, the distribution of DiR@TPGS-PBTE NPs in healthy mice showed no obvious difference from free Dir and DiR@TPGS-PBTE NPs, but was stronger and wider than that of the other two in DSS-induced mice. This is consistent with the *in vitro* drug release results.

Further studies were performed to explore the distribution of nanoparticles in specific organs. At 12 ​h, after the mice were given free DiR, DiR@TPGS-PBTE NPs and DiR@TPGS-PLGA NPs, their heart, liver, spleen, lung, kidney, colon and peripheral blood were removed at each time point for *in vitro* imaging. As shown in Fig. 3J, the fluorescence intensity of free DiR and DiR@TPGS-PBTE NPs in the colon of the colitis mice was stronger than that of normal mice; while the distribution of DiR@TPGS-PLGA NPs in the colon of the healthy mice was close to that of the colitis mice. In line with this observation, quantification analysis showed relatively higher fluorescence intensity of DiR@TPGS-PBTE NPs-treated colitis colon than that of healthy colon (Fig. 3K, M). Besides, the area under the fluorescence intensity-time curve (AUC) was calculated. In colitis colon, we found that the AUC of DiR@TPGS-PBTE NPs was 1.3- and 2.3-fold higher than that of free DiR and DiR@TPGS-PLGA NPs, respectively (Fig. 3L, N); its AUC improved 16 times more than that of the healthy colon. This confirms the super ability of TPGS-PBTE NPs in targeting colitis colon. In normal colon, the AUC of DiR@TPGS-PLGA NPs was 1.4- and 7.8-fold higher than that of free DiR and DiR@TPGS-PBTE NPs, respectively (Fig. 3L); the value was almost the same as that of UC mice. Also, the frozen sections of the colons were observed by fluorescence microscope (Fig. 3O). Fluorescence quantitative analysis showed that the DiR intensity of DiR@TPGS-PBTE NPs-treated colitis colon was 1.7- and 2.9- fold higher than that of free DiR and DiR@TPGS-PLGA NPs-treated colitis colon, and also 4.4-fold higher than that of DiR@TPGS-PBTE NPs-treated normal colon, respectively (Fig. 3P). Also, there was obvious accumulation of DiR in the liver, spleen and peripheral blood of the colitis mice (Fig. 3B and E). Weak fluorescence appeared in the healthy mice. Since DSS-induced colitis developed mucosal damage, the more DiR was released, the more DiR crossed the enteric-blood barrier to reach the metabolic organs. After 12 ​h of treating the mice via gavage, the average fluorescence intensity of DiR@TPGS-PBTE NPs in the liver of the colitis mice was 4.2- and 13.5-fold higher than that of free DiR and LUT@TPGS-PLGA NPs, respectively (Fig. 3C). The fluorescence intensity of DiR@TPGS-PBTE NPs in the spleen of colitis mice was 2.0- and 3.9-fold higher than that of free DiR and LUT@TPGS-PLGA NPs, respectively (Fig. 3D). Likewise, quantitative analysis showed higher fluorescence intensity of DiR@TPGS-PBTE NPs in the blood of the colitis mice compared to the healthy mice (Fig. 3F and H), and its AUC was 2.5- and 2.8-fold higher than that of free DiR and LUT@TPGS-PLGA NPs, respectively (Fig. 3I). In contrast, the AUC of DiR@ TPGS-PLGA NPs in the blood of the normal mice was 4.2- and 4.6-fold higher than that of free DiR and LUT@ TPGS-PBTE NPs, respectively (Fig. 3G).

Altogether, these results demonstrated that TPGS-PBTE NPs could selectively accumulate in the inflamed colon tissues with stronger diffusion ability than the control TPGS-PLGA NPs. When loaded into TPGS-PBTE NPs, the payload drugs can be effectively delivered into the colitis tissue, thereby maximizing bioavailability and enhancing therapeutic effects.

### 3.5 Cell uptake

**Fig. 4:**
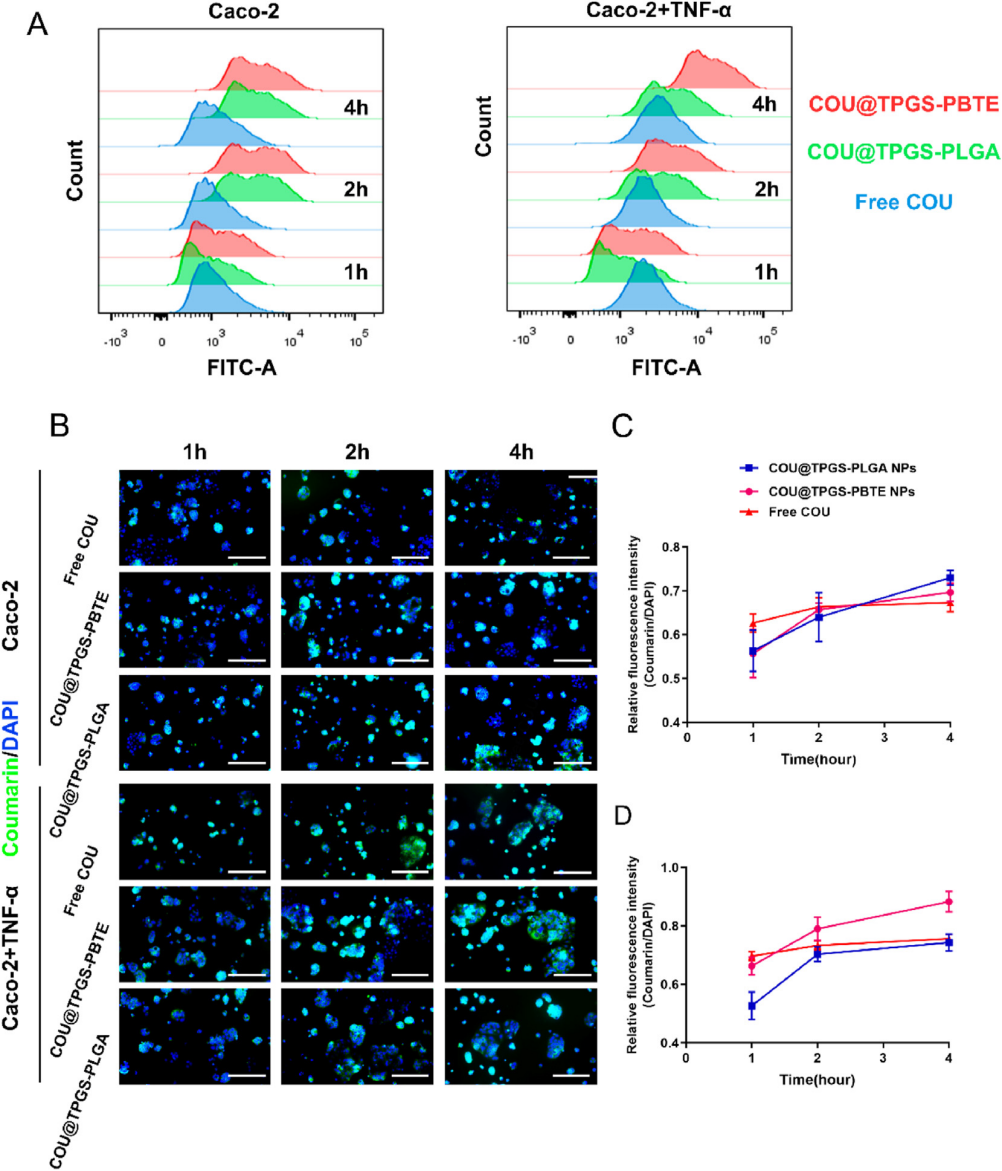
Cell uptake of COU@TPGS-PBTE NPs. A) Flow cytometry analysis of Caco-2 ​cells treated with free coumarin-6, COU@TPGS-PBTE NPs and COU@TPGS-PLGA NPs for 1,2,4 ​h. B) Fluorescence microscope images of Caco-2 ​cells treated with COU@TPGS-PBTE NPs and COU@TPGS-PLGA NPs for 1,2,4 ​h, the scale bar represents 100 ​μm. C) Quantitative analysis of fluorescence intensity in normal Caco-2 ​cells. D) Quantitative analysis of fluorescence intensity in TNF-α pretreated Caco-2 ​cells. Data were expressed as mean ​± ​SD.

In order to evaluate the cell uptake behavior of the nanoparticles, Caco-2 ​cells were stimulated with/without TNF-α for 24 ​h, and then cultured with free coumarin-6, COU@TPGS-PBTE NPs and COU@TPGS-PLGA NPs for 1, 2 and 4 ​h, respectively. Flow cytometry and fluorescence microscopy were used to observe the intracellular fluorescence intensity of coumarin-6. The nucleus was stained with DAPI. As shown in Fig. 4A, in normal Caco-2 ​cells, all the three treated groups showed similar fluorescence intensity during the period. In contrast, after pretreated with TNF-α for 24 ​h (inflamed Caco-2 ​cells), COU@TPGS-PBTE NPs had stronger fluorescence intensity than COU@TPGS-PLGA NPs and free coumarin-6. Immunofluorescence images further verified this result (Fig. 4B). After 4 ​h of incubation in TNF-α pretreated Caco-2 ​cells, the fluorescence intensity of COU@TPGS-PBTE NPs was 1.2 times higher than that of COU@TPGS-PLGA NPs and free coumarin-6 (Fig. 4D). While in normal Caco-2 ​cells, quantitative analysis of fluorescence intensity showed no significant difference in the three groups (Fig. 4C). It is also interesting to see that the fluorescence intensity of COU@TPGS-PLGA NPs in normal Caco-2 ​cells and inflamed Caco-2 ​cells was almost the same, while that of COU@TPGS-PBTE NPs in inflamed Caco-2 ​cells was ∼1.3 times higher compared to normal Caco-2 ​cells. These results indicated the enhanced drug delivery capability of ROS-responsive TPGS-PBTE NPs to inflamed cells.

### 3.6 Therapeutic effects of LUT@TPGS-PBTE NPs on DSS-induced acute colitis mice

**Fig. 5:**
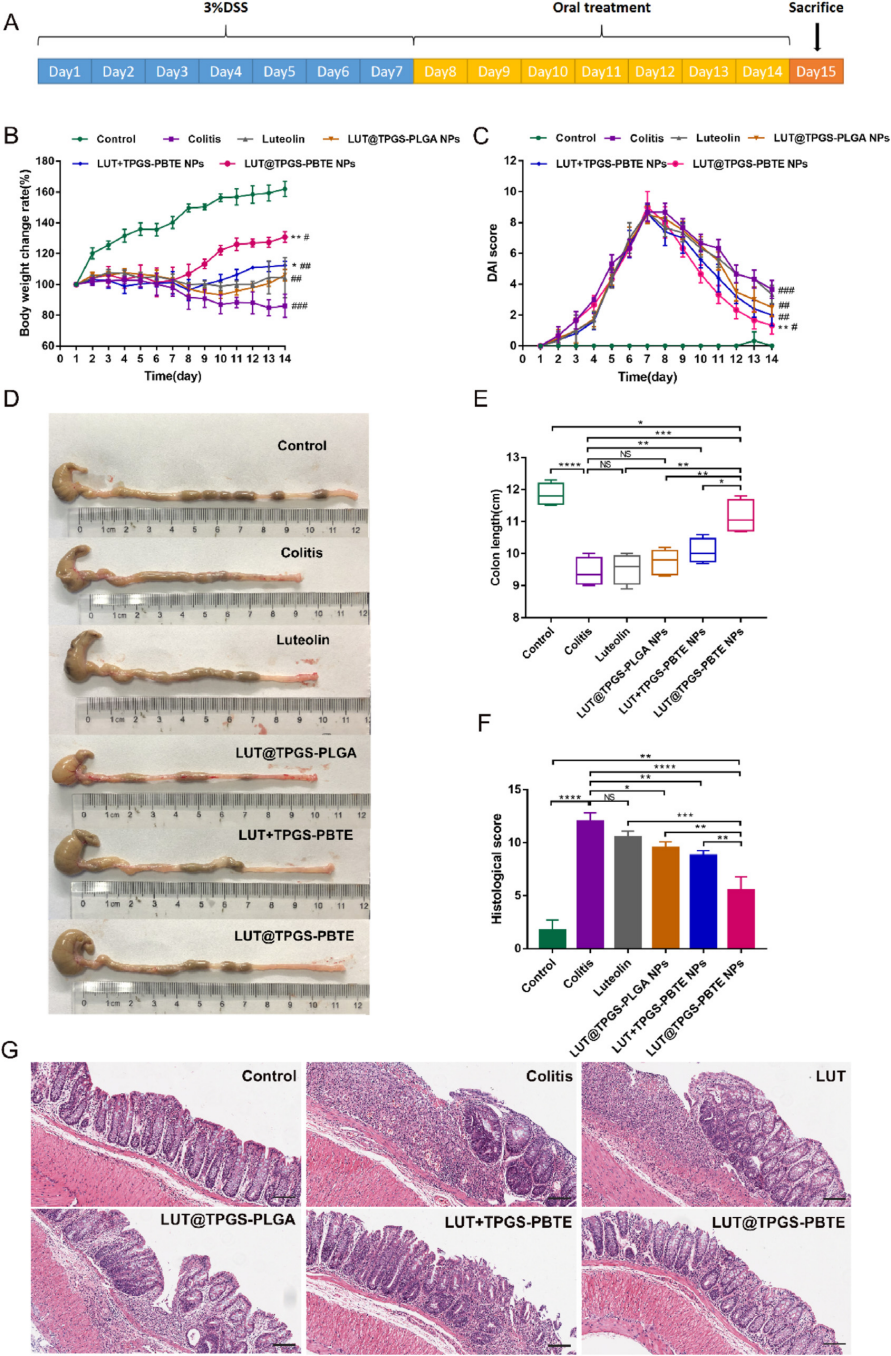
Therapeutic effects of LUT@TPGS-PBTE NPs on acute colitis in mice. A) Schematic illustration of acute colitis model and treatment. B) Changes of bodyweight. C)DAI score. D) Representative photograph of colons. E) Quantitative analysis of colon length. F) Histological score of H&E staining. G) H&E staining of colonic tissue. The scale bar represents 100 ​μm. Data were expressed as mean ​± ​SD. #, ∗p ​< ​0.05, ##, ∗∗p ​< ​0.01, ###, ∗∗∗p ​< ​0.001, ####, ∗∗∗∗p ​< ​0.0001; NS, no significance. ∗ vs DSS group, # vs Control group.

Since orally administered TPGS-PBTE NPs may preferentially accumulate in the inflamed colon, and release its payload, we reasonably hypothesize that TPGS-PBTE NPs can serve as an ideal drug delivery system for the targeted treatment of colitis. Firstly, acute colitis models were induced in mice through administration of 3% DSS for 7 days. Then the mice were treated with saline, free LUT, LUT@TPGS-PLGA NPs, LUT ​+ ​TPGS-PBTE NPs and LUT@TPGS-PBTE NPs by gavage for another 7 days, respectively (Fig. 5A). The dosage of LUT in each group was 20 ​mg/kg. TPGS-PLGA NPs was utilized to prove the advantages of ROS-responsive TPGS-PBTE NPs. During the experiment, the bodyweight of animals in DSS group gradually decreased within 14 days (Fig. 5B). Compared with the DSS group, the bodyweight loss dramatically improved in mice treated with LUT@TPGS-PBTE NPs (P ​< ​0.01) and LUT ​+ ​TPGS-PBTE NPs (p ​< ​0.05). However, there was no significant difference of weight improvement in free LUT and LUT@TPGS-PLGA NPs groups. Furthermore, the DAI score, assessing the severity of colitis, distinctly increased after 7 days of DSS treatment (Fig. 5C). On day 14, after the mice were treated with the various drugs via oral route, it was markedly reduced in LUT@TPGS-PBTE NPs group (P ​< ​0.01). The length of colon in the colitis group was significantly shorter than that in normal group, which was consistent with the clinical manifestations of bloody diarrhea and weight loss (Fig. 5D and E). The colon shortening could be rescued by LUT@TPGS-PBTE NPs (p ​< ​0.001) and LUT ​+ ​TPGS-PBTE NPs (p ​< ​0.01) therapies. H&E staining was further applied to analyze the pathological changes of colon tissue sections. As shown in Fig. 5G, epithelial barrier defect, crypt damage, depletion of goblet cells and inflammatory cell infiltration were observed in the colitis group, which was in sharp contrast with the normal group. The free LUT treated group exhibited serious crypt destruction, partial epithelial damage and plenty of inflammatory cell infiltration because LUT (in such dosage) had a weak treatment effect on DSS-induced colitis. The colonic pathological changes of the other treated groups were all alleviated in different degrees, with distinctively reduced histological score (Fig. 5F). In LUT@TPGS-PBTE NP treated groups, their tissue structure was rescued, crypt and goblet cells returned to normal, and little inflammatory cell infiltration was observed.

**Fig. 6:**
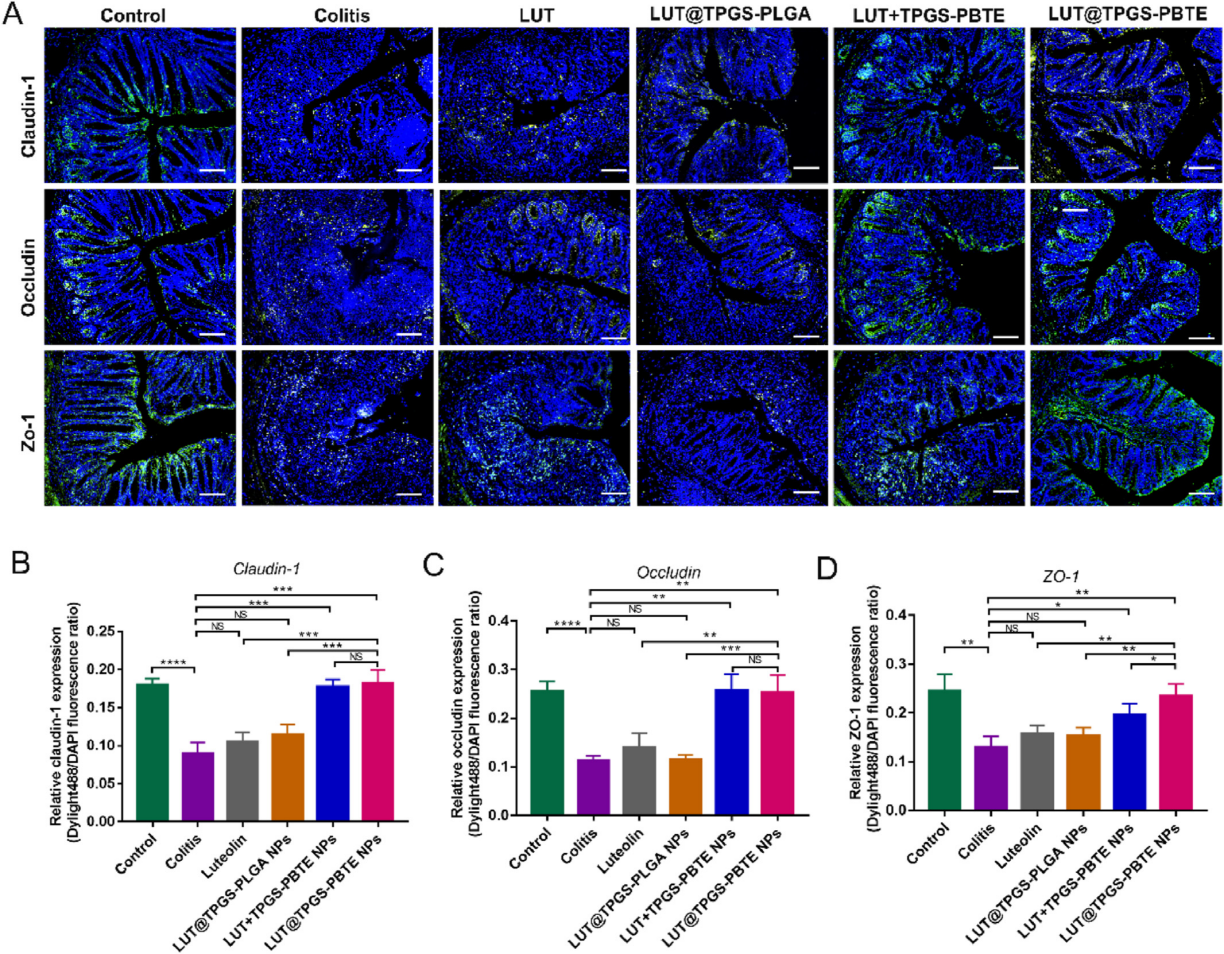
Immunofluorescence assay presented the expression of TJ proteins in mice colon. A) Representative immunofluorescence staining images of claudin-1, occludin and ZO-1, the scale bar represents 100 ​μm. Green signal represents the target protein. DAPI stands for nuclear staining. B) Quantitative analysis of the fluorescence intensity of C) claudin-1. D) occludin. E) ZO-1. Data were expressed as mean ​± ​SD. ∗p ​< ​0.05, ∗∗p ​< ​0.01, ∗∗∗p ​< ​0.001, ∗∗∗∗p ​< ​0.0001; NS, no significance. (For interpretation of the references to color in this figure legend, the reader is referred to the Web version of this article.)

One of the most striking pathological features of DSS-induced colitis is the destruction of intestinal epithelial tight junction proteins. Tight junction (TJ) proteins, mainly including occludin, claudins and zonulaoccludens (ZO), help to maintain the intestinal barrier function [39]. To determine the repair effect of the formulations on intestinal barrier, immunofluorescence staining was utilized to detect the expression of claudin-1, occludin and ZO-1 in the colons of the mice (Fig. 6A). We observed that the fluorescence intensity of the three proteins was clearly diminished in the DSS treated mice (Fig. 6B–D). And their depletion was restored by LUT ​+ ​TPGS-PBTE NPs and LUT@TPGS-PBTE NPs. In the LUT@TPGS-PBTE NPs group, the fluorescence intensity was nearly similar to that of the normal group. Further, we conducted western blotting assay to detect the occluding and ZO-1 protein levels in Caco-2 ​cells (Figure S5A). quantitative analysis (Figure S5B-C) shows that all the formulations have repaired the tight junction proteins of Caco-2 ​cells after TNF-α injury in different degrees. LUT ​+ ​TPGS-PBTE NPs and LUT@TPGS-PBTE NPs have more obvious repair effects. These results suggest that LUT@TPGS-PBTE NP alleviates DSS-induced colitis partly by promoting intestinal mucosal barrier repair.

### 3.7 LUT@TPGS-PBTE NPs regulate CD4^+^ T lymphocyte subsets balance in colitis mice

**Fig. 7:**
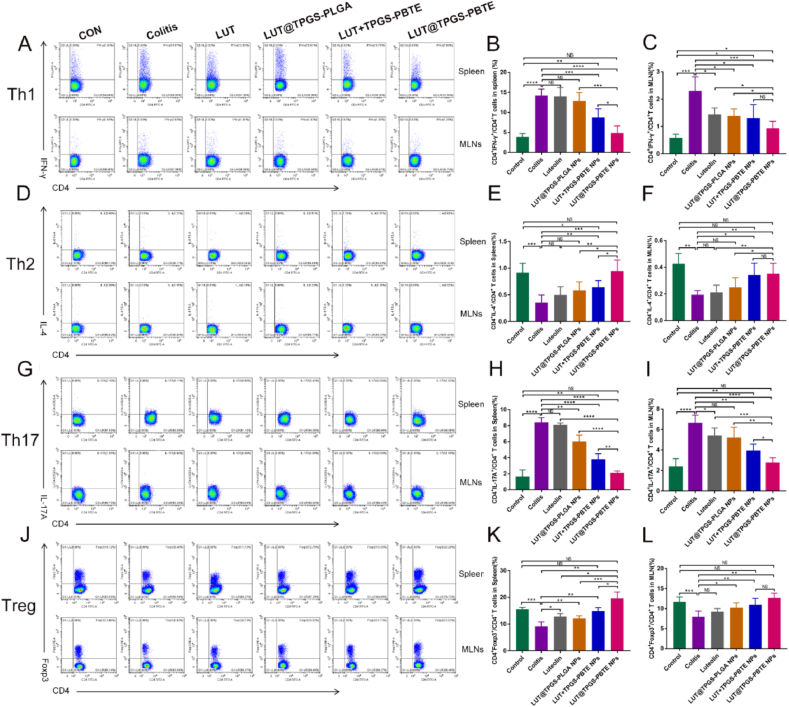
Capacity of immune regulation of LUT@TPGS-PBTE NPs on acute colitis in mice. A) Representative flow cytometric profiles of Th1 cell in spleen and MLNs. B) Quantitative analysis of Th1 percentage in spleen. C) Quantitative analysis of Th1 percentage in MLNs. D) Representative flow cytometric profiles of Th2 cell in spleen and MLNs. E) Quantitative analysis of Th2 percentage in spleen. F) Quantitative analysis of Th2 percentage in MLNs. G) Representative flow cytometric profiles of Th17 ​cell in spleen and MLNs. H) Quantitative analysis of Th17 percentage in spleen. I) Quantitative analysis of Th17 percentage in MLNs. J) Representative flow cytometric profiles of Treg in spleen and MLNs. K) Quantitative analysis of Treg percentage in spleen. L) Quantitative analysis of Treg percentage in MLNs. Data were expressed as mean ​± ​SD. ∗p ​< ​0.05, ∗∗p ​< ​0.01, ∗∗∗p ​< ​0.001, ∗∗∗∗p ​< ​0.0001; NS, no significance.

Infiltration of immune cells especially CD4^+^ T lymphocytes in the colon and immune organs is a hallmark of UC. Th1 cells secrete proinflammatory factors such as IFN-γ and TNF-α to aggravate intestinal inflammation. Th2 cells mainly secrete immune effector molecules such as IL-4, IL-5 and IL-10, which can induce the production of IgA type B cells, and then release a large amount of IgA to protect intestinal mucosa. Th17 ​cells can release IL-17, IL-22, IL-21, granulocyte-macrophage colony stimulating factor (GM-CSF) and other proinflammatory factors. They can recruit neutrophils and mononuclear macrophages to the inflamed colon, thus aggravating UC [40]. Tregs inhibit Th17 differentiation and IL-17A expression partially by releasing IL-10 [41]. To investigate the potential mechanism by which LUT@TPGS-PBTE NPs therapy mitigate intestinal inflammation and improve mucosa barrier function, we analyzed the CD4^+^ T lymphocyte subsets in both spleen and mesenteric lymph nodes by flow cytometry. Fig. 7A presents the percentage of Th1 cells in CD4^+^ T lymphocytes. Quantitative analysis showed distinctively higher percentage of Th1 cells in both the spleen and MLNs of the colitis group compared to the control group (Fig. 7B and C). In the spleen, the amount of Th1 cells could hardly be reversed by free LUT or LUT@TPGS-PLGA NPs therapies. However, LUT ​+ ​TPGS-PBTE NPs and LUT@TPGS-PBTE NPs significantly reduced the percentage of Th1 cells. Among the groups, the LUT@TPGS-PBTE NPs group exhibited the best Th1 inhibiting effect (Fig. 7B). In MLNs, all of the formulations showed an obvious reduction of Th1 percentage, while LUT@TPGS-PBTE NPs had the lowest amount of Th1 cells (Fig. 7C). Th2 cells, another critical subset of CD4^+^ T cells, significantly declined in the saline-treated group, in both their spleen and MLNs (Fig. 7D–F). Quantitative analysis showed that LUT ​+ ​TPGS-PBTE NPs and LUT@TPGS-PBTE NPs therapies could effectively elevate the amount of Th2 cells in both spleen and MLNs, while LUT@TPGS-PBTE NPs increased it more significantly. Presentative photograph of percentage of Th17 ​cells in CD4^+^ T lymphocytes is displayed in Fig. 7G. In line with previous studies, the percentage of Th17 ​cells in both spleen and MLNs drastically increased in the saline-treated group (Fig. 7H and I). In the spleen, except in the LUT group, the other formulations had lower percentage of Th17 ​cells compared to the colitis group (Fig. 7H). In the MLNs only the LUT@TPGS-PLGA NPs group had no difference in Th17 ​cells in comparison with the colitis group (Fig. 7I). Among all the treatments, LUT@TPGS-PBTE NPs most effectively reduced the amount of Th17 ​cells. The changes of Tregs in the spleen and MLNs were also analyzed (Fig. 7J). The percentage of Tregs in CD4^+^ T lymphocytes significantly decreased in the colitis mice compared to the control group in both spleen and MLNs. In the spleen, all treatment groups showed an increased percentage of Tregs (Fig. 7K). However, only LUT group exhibited no significant difference in Tregs with colitis group in their MLNs (Fig. 7L). Of all the therapies, LUT@TPGS-PBTE NPs most effectively elevated the amount of Tregs. Taken together, these results demonstrated that LUT@TPGS-PBTE NPs could resolve inflammation of the colons and accelerate wound healing partly via regulating the balance of CD4^+^ T lymphocyte subsets.

**Fig. 8:**
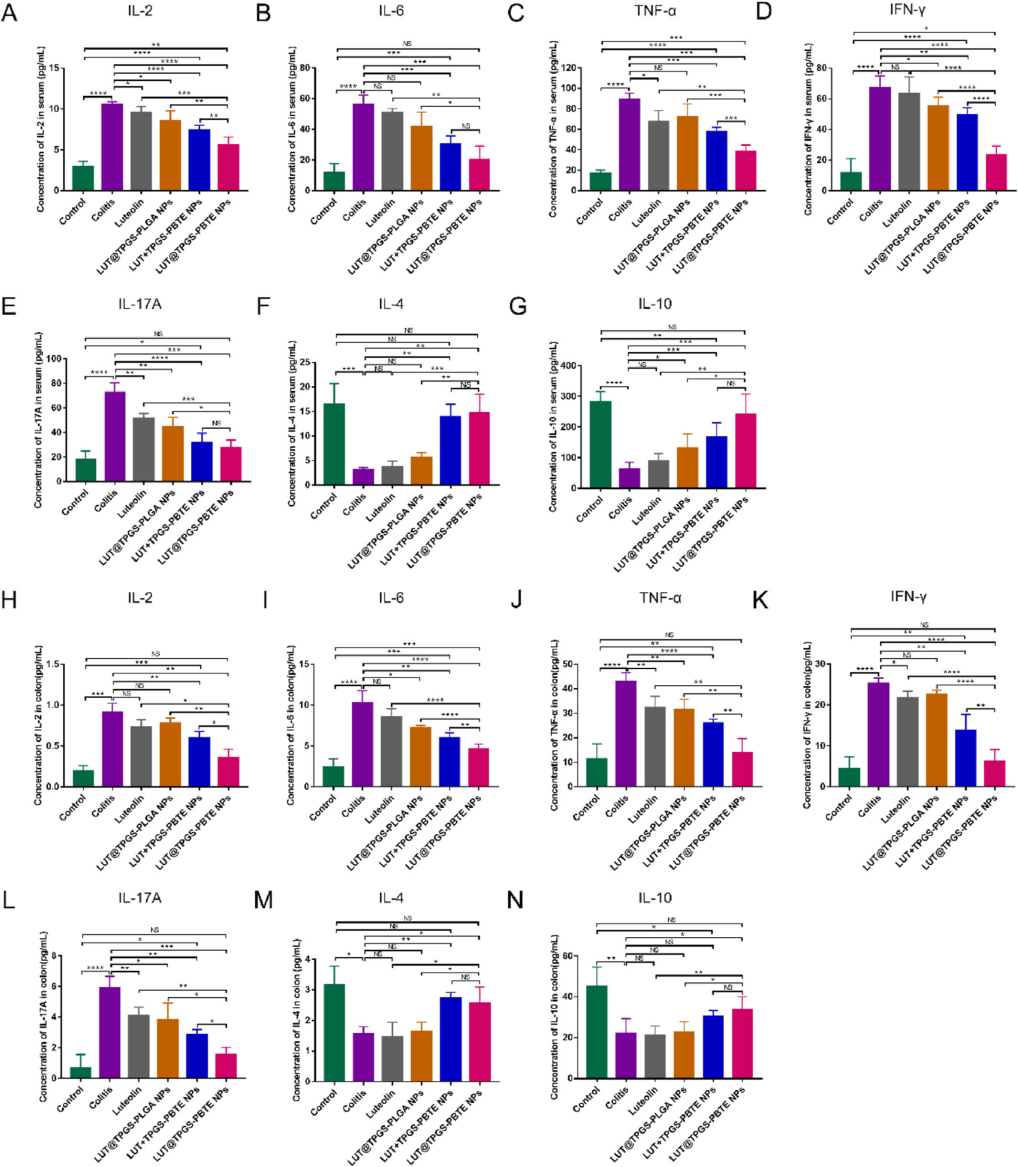
Cytometric Bead Array analysis of Th1/Th2/Th17 associated cytokines. A) IL-2 level in serum. B) IL-6 level in serum. C) TNF-α level in serum. D) IFN-γ level in serum. E) IL-17A level in serum. F) IL-4 level in serum. G) IL-10 level in serum. H) IL-2 level in colon. I) IL-6 level in colon. J) TNF-α level in colon. K) IFN-γ level in colon. L) IL-17A level in colon. M) IL-4 level in colon. N) IL-10 level in colon. Data were expressed as mean ​± ​SD. ∗p ​< ​0.05, ∗∗p ​< ​0.01, ∗∗∗p ​< ​0.001, ∗∗∗∗p ​< ​0.0001; NS, no significance.

To further confirm the CD4^+^ T lymphocyte subsets regulation by LUT@TPGS-PBTE NPs, Th1/Th2/Th17 associated cytokines in the colonic tissues and serum were also analyzed by CBA. Quantitative analysis showed that the proinflammatory factors secreted by Th1/Th2/Th17, including IL-2, IL-6, TNF-α, IFN-γ and IL-17A significantly increased in the colitis group in both their serum and colonic tissues (Fig. 8A–E, H-L). Although the proinflammatory factors mentioned above were reduced by all the formulations in different degrees, LUT@TPGS-PBTE NPs most effectively declined their expression. Especially the concentration of IL-6 and IL-17A in the serum, IL-2, TNF-α, IFN-γ and IL-17A in the colon showed no difference between LUT@TPGS-PBTE NPs and normal groups. In addition, the anti-inflammatory cytokine IL-4, IL-10 secreted by Th2 cells showed a distinctive reduction in the serum and colonic tissues of the colitis mice (Fig. 8F–G, M−N). Among the formulations, LUT ​+ ​TPGS-PBTE NPs and LUT@TPGS-PBTE NPs significantly rescued the concentration of the two cytokines, and LUT@TPGS-PBTE NPs had no significant difference from the normal mice.

### 3.8 LUT@TPGS-PBTE NPs alleviate oxidative stress in the inflamed colon

**Fig. 9:**
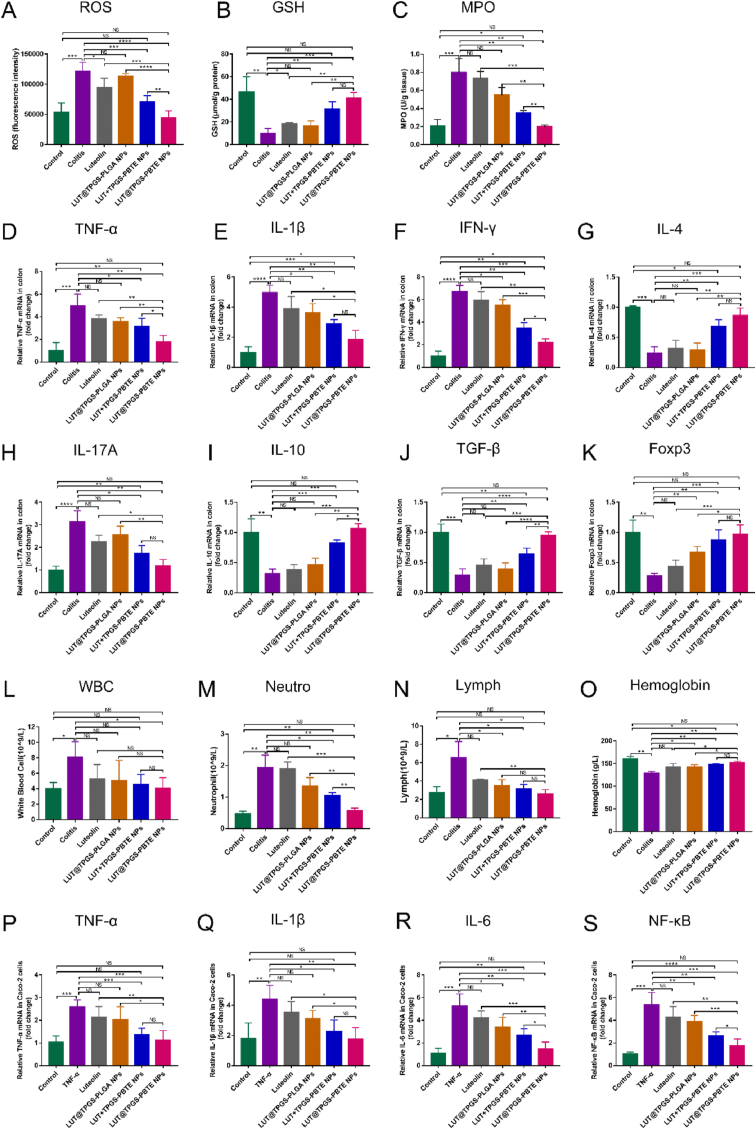
Antioxidant and anti-inflammatory effects of DiR@TPGS-PBTE NPs on acute colitis in mice. A) ROS level in colon. B) GSH level in colon. C) MPO activity in colon. Quantitative reverse-transcriptase polymerase chain reaction (qRT-PCR) analysis of D) TNF-α, E) IL-1β, F) IFN-γ, G) IL-4, H) IL-17A, I) IL-10, J) TGF-β, and K) Foxp3 in colon. Blood routine examination of L) White blood cell, M) Neutrophils, N) Lymphocyte and O) Hemoglobin. qRT-PCR analysis of P) TNF-α, Q) IL-1β, R) IL-6 and S) NF-κB in Caco-2 ​cells. Data were expressed as mean ​± ​SD. ∗p ​< ​0.05, ∗∗p ​< ​0.01, ∗∗∗p ​< ​0.001, ∗∗∗∗p ​< ​0.0001; NS, no significance.

Oxidative Stress (OS) refers to a state of imbalance between oxidation and antioxidant effects in the body, which leads to neutrophil inflammatory infiltration, increased protease secretion and the production of a large number of oxidative intermediates. ROS, including superoxide anion, hydroxyl free radical and hydrogen peroxide, etc. is an indicator of tissue oxidative stress level. To test the ROS-eliminating capacity of LUT@TPGS-PBTE NPs as well as the other formulations, we further assessed ROS concentration in colons. As shown in Fig. 9A, the ROS level in the colitis group significantly increased and was 2.2-fold higher than that in normal mice, while except LUT@TPGS-PLGA NPs, the other formulations greatly reduced the ROS level in different degrees. LUT@TPGS-PBTE NPs had the lowest ROS activity which was almost equal to that of the healthy group. GSH, as an important antioxidant and free radical scavenger in the body, reflecting the antioxidant ability of the drugs, was measured as well. Consistent with previous literature [22], there was obvious reduction of GSH expression in the colon of DSS-induced colitis (Fig. 9B); it was significantly increased by all treatment groups except LUT@TPGS-PLGA NPs. LUT@TPGS-PBTE NPs greatly elevated GSH concentration in the colon, which was 4.1-fold higher than that in colitis mice. MPO, a peroxidase secreted by infiltrating neutrophils, serving as an effective marker of both oxidative stress and inflammation, was also detected. The activity of MPO in the colitis mice was much more than that in normal mice, and was significantly improved by LUT ​+ ​TPGS-PBTE NPs and LUT@TPGS-PBTE NPs therapies. Especially in LUT@TPGS-PBTE NPs group, the value was the lowest one (0.19 ​± ​0.01) and showed no significance compared to healthy mice (0.20 ​± ​0.03) (Fig. 6C). These results demonstrate the excellent ability of LUT@TPGS-PBTE NP to eliminate reactive oxygen species and as well as its antioxidation ability.

### 3.9 Anti-inflammatory effects of LUT@TPGS-PBTE NPs

To further gain insight into the colonic and systemic anti-inflammatory capacity of LUT@TPGS-PBTE NPs in regulating the expression of proinflammatory and anti-inflammatory factors, Quantitative reverse-transcriptase polymerase chain reaction analysis was also performed. Compared with the normal group, TNF-α, IL-β, IFN-γ and IL-17A obviously increased in colitis tissues (Fig. 9D–F, H). And the above proinflammatory cytokines were significantly decreased by LUT ​+ ​PGS-PBTE NPs and LUT@TPGS-PBTE NPs. In LUT@TPGS-PBTE NPs group, the mRNA level of TNF-α and IL-17A was not different from that of the normal group. On the contrary, the expression of anti-inflammatory factors IL-4, IL-10, TGF-β and Foxp3 were significantly declined in the colitis group compared to the healthy mice (Fig. 9G, I-K). LUT ​+ ​PGS-PBTE NPs and LUT@TPGS-PBTE NPs therapies remarkably increased these factors and the levels of LUT@TPGS-PBTE NPs were similar to that of the normal group. These results confirm that LUT@TPGS-PBTE NPs could inhibit the overwhelming inflammatory response, partly responsible for the treatment of colitis. In addition, we also performed routine blood tests on the mice. As shown in Fig. 9L–N, the colitis mice had significantly elevated levels of white blood cells, neutrophils, and lymphocytes in their blood, suggesting systemic inflammation. All the formulations have been shown to reduce the amounts of these cells to varying degrees except for LUT, and LUT@TPGS-PBTE NPs was the most effective. Besides, the hemoglobin, reflecting the anemia degree of the mice after hematochezia, decreased in the colitis mice (Fig. 9O). LUT@TPGS-PBTE NPs rescued the hemoglobin of mice with colitis and showed no significant difference from the normal group.

Anti-inflammatory mechanism of LUT@TPGS-PBTE NPs was further investigated in Caco-2 ​cells *in vitro*. Epithelial NF- κ B signaling pathway maintains intestinal homeostasis by regulating the proliferation, survival and apoptosis of intestinal epithelial cells. Both inactivation and hyperactivation of this pathway predispose to intestinal inflammation [42]. To investigate the NF-κB signaling pathway regulation capacity of LUT@TPGS-PBTE NPs on intestinal epithelial cells, Caco-2 ​cells were stimulated with TNF-α(100 ​ng/ml) for 24 ​h to establish an *in vitro* inflammatory model. Fig. 9P–S shows the results of qRT-PCR analysis of NF-κB and its downstream inflammatory factors. There was significantly increased level of TNF-α, IL-1β, IL-6 and NF-κB in TNF-α-induced Caco-2 ​cells, suggesting enhanced inflammation. Under the treatments of LUT@TPGS-PLGA NPs and LUT ​+ ​TPGS-PBTE NPs, the concentration of IL-6 and NF-κB was remarkably reduced to some extent. Only LUT ​+ ​TPGS-PBTE NPs and LUT@TPGS-PBTE NPs therapies could effectively decrease the level of TNF-α and IL-1β. Moreover, among the formulations, LUT@TPGS-PBTE NPs group had the lowest level of proinflammatory factors, with nearly no difference compared to normal Caco-2 ​cells. Combined, ex vivo results further confirm the anti-inflammatory effect of LUT@TPGS-PBTE NPs.

### 3.10 Safety evaluation

**Fig. 10:**
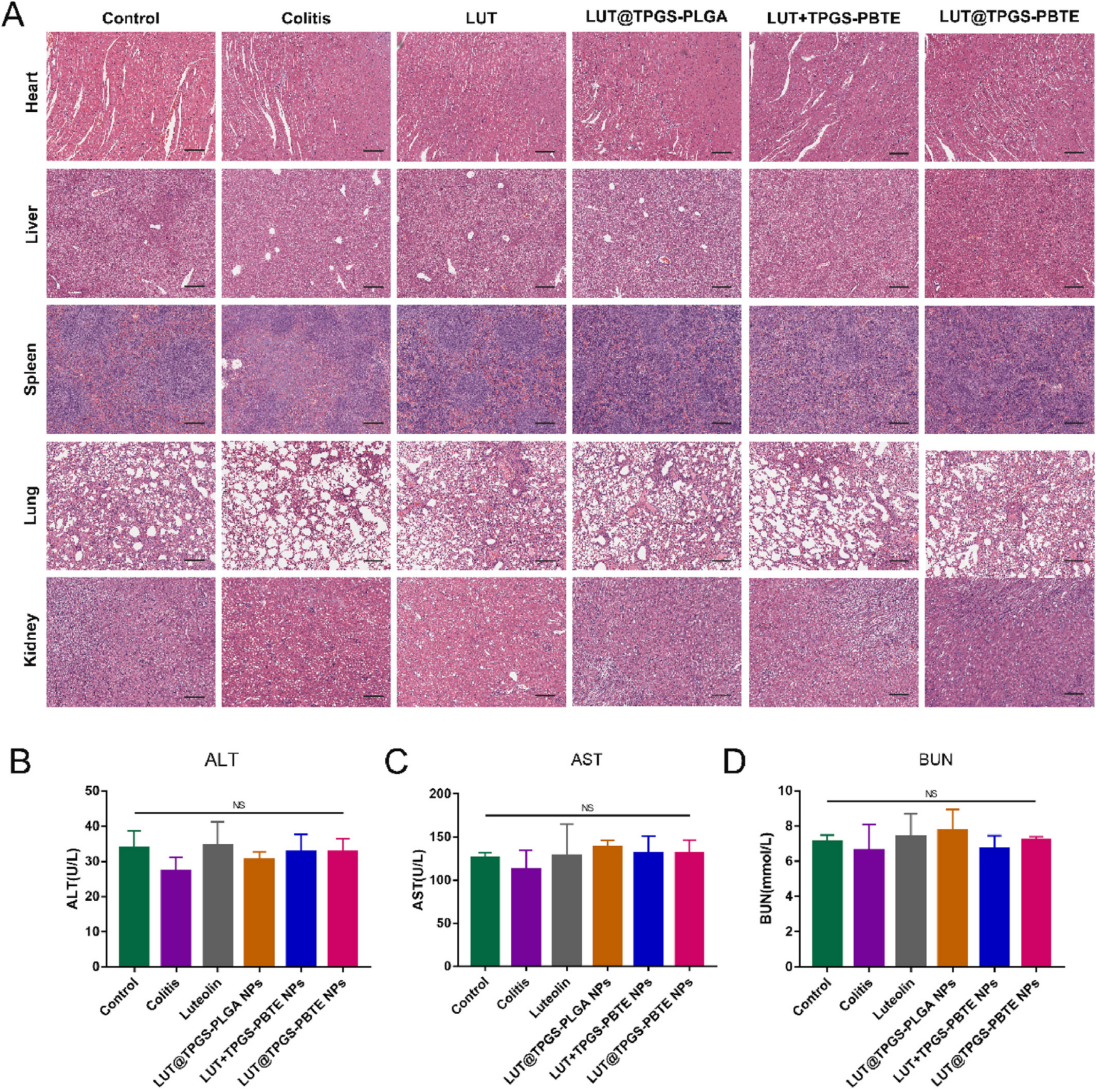
Safety evaluation. A) H&E staining of heart, liver, spleen, lung and kidney. The scale bar represents 100 ​μm. Blood biochemical test of B) ALT, C) AST and D) BUN. NS, no significance.

To assess the biosafety of the formulations, H&E staining was adopted to determine whether there were pathological changes in the main organs, including the heart, liver, spleen, lungs and kidneys of the mice after the whole period of treatment. As shown in Fig. 10A, no obvious pathological changes were found in the pathological sections of the heart, liver, spleen, lungs and kidneys in each group. And in the blood biochemical test of ALT, AST and BUN (Fig. 10B–D), indicators of liver and kidney function, showed no difference from healthy mice. These results suggest that LUT@TPGS-PBTE NPs exhibits excellent safety profiles via oral administration.

## 4 Discussion

Ulcerative colitis (UC) is one of the two major forms of inflammatory bowel disease (IBD), characterized by mucosal inflammation that starts in the rectum and extends to the proximal colon continuously [43,44]. Although the pathogenesis is still under study, numerous studies support that excessive immune activation beneath the intestinal epithelium as well as overwhelming mucosal inflammation is closely related to its pathogenesis of UC [45]. Abnormal immunity of intestinal mucosa includes innate and adaptive immunity. Disorders of adaptive immunity, mainly mediated by T cells, with fundamental defects of CD4^+^ helper T [Th] cells and regulatory T [Treg] cells, are considered predominant in the mechanism of UC [46]. CD4^+^ T cells primarily include IFN-γ-expressing Th1 cells, IL-4-secreting Th2 cells, Th17 ​cells that express IL-17A, IL-17F and IL-22 and IL-10-secreting Treg cells with high Foxp3 expression. Reactive oxygen species (ROS) refers to the general term for oxygen-containing free radicals related to oxygen metabolism and peroxides that are easy to form free radicals in the body. Oxidative metabolism in the body can continuously form reactive oxygen species, which have positive physiological effects in a certain stage. Oxidative stress (OS), caused by an imbalance between ROS production and ROS clearance by the detoxification mechanisms of biological systems, has been recognized as a common mechanism in UC [13,14]. Overexpression of ROS is positively associated with aggravated inflammatory manifestations in the gut of UC patients and DSS induced mice. OS is not only the product of inflammatory cells metabolism, but also the cause of aggravating inflammatory infiltration of neutrophils. Besides, study has confirmed that reactive oxygen species participate in the activation of lymphocytes and promote the proliferation of T cells, thereby aggravating the immune response of colitis [47]. Literature have reported that oxidative stress plays a vital role in the reduction of Treg cells, which can suppress excessive immune response in UC [[48], [49], [50]]. In vivo experiments have demonstrated that either antioxidants or free radical scavengers are an effective targeted therapy for UC enteritis [15,16].

In this work, we designed a ROS-responsive drug carrier, TPGS-PBTE, to develop a safe and efficient formulation against UC via the synergistic effect of ROS cleavage and ROS-responsive drug targeting release. Thioether is a usually used ROS-responsive group which can be oxidized to sulfoxide [19], inducing hydrophilic-hydrophobic transition and increased hydrolysis ratio of sulfoxide adjacent ester bond. We first tested the ROS responsiveness of TPGS-PBTE NPs *in vitro*. In the presence of hydrogen peroxide, after 48 ​h of incubation, the diameter of both TPGS-PBTE NPs and LUT@TPGS-PBTE NPs gradually increased and aggregations can be observed, indicating the outstanding profiles of their ROS-responsiveness. While the diameter of TPGS-PLGA NPs and LUT@TPGS-PLGA NPs almost remained unchanged. *In vitro* drug release experiment also confirmed that the accelerated LUT release rate from LUT@TPGS-PBTE NPs stimulated with hydrogen peroxide was 1.5-fold higher than that from LUT@TPGS-PBTE NPs without hydrogen peroxide stimulation, and the LUT release rate from LUT@TPGS-PLGA NPs showed nonsignificant difference either with or without hydrogen peroxide (Fig. 2E). ^1^H NMR and MS results confirmed the formation of sulfoxide and hydrolysis after incubation in 1 ​mM ​H_2_O_2_ (Figure S2). We also investigated the reactive oxygen species scavenging capability of LUT@TPGS-PBTE NPs *in vitro via* Caco-2 inflammatory model. As shown in Figure S4, TNF-α treatment significantly elevates the ROS level of Caco-2 ​cells, and all the formulations exhibits significant reduction of ROS, especially the LUT@TPGS-PBTE NPs group, which has the most pronounced reduction. As proved by in and ex vivo imaging results (Fig. 3A, J and O), excellent ROS-responsive TPGS-PBTE NPs had well targeting efficiency on the inflamed colon. DiR@TPGS-PBTE NPs showed drastically higher fluorescence intensity in inflamed colon, which was 16-fold higher than that in healthy colon (Fig. 3L and N). However, the non-ROS responsive DiR@TPGS-PLGA NPs showed no significant difference of fluorescence intensity between healthy and inflamed colon, indicating that the hydrolysis and drug release properties of TPGS-PLGA NPs could not be affected under oxidative stress. All these results demonstrate that due to excellent ROS responsiveness, TPGS-PBTE NPs can precisely target the inflamed colon, thus they can be a potential strategy of UC therapy.

Furthermore, we were pleasantly surprised to find that, as a ROS responsive material, TPGS-PBTE NPs themselves could relieve UC. Compared with LUT therapy, LUT ​+ ​TPGS-PBTE NPs showed better capability of scavenging reactive oxygen species. TPGS-PBTE NPs could reduce the ROS concentration and MPO amount in colitis tissue, and promote GSH concentration (Fig. 9A–C), thus rebuilding antioxidant/oxidation balance in the colons. Considering that reactive oxygen species are pathological manifestations of UC colon, ROS-scavenging strategy may play an important supporting role and achieve well synergistic effect with anti-inflammatory drugs in UC treatment.

Immune response caused by intestinal injury and overexpression of inflammatory cytokines play an important role in UC [51,52]. Since Mosmann et al. [53] found that CD4^+^ T cells can be divided into Th1 and Th2 types according to the cytokines they secreted in 1986, people have increasingly studied the relationship between Th1/Th2 cell imbalance in autoimmune diseases. Th1 secretes IFN- γ, TNF-α, IL-2 and other pro-inflammatory factors, Th2 secretes IL-4, IL-10, IL-13 and other anti-inflammatory factors. Studies have found that the abnormality of Th1/Th2 cell balance is one of the important immune factors in the pathogenesis of inflammatory bowel disease [54]. Th17 ​cells, induced by TGF-β and IL-6 and identified by the secretion of amounts of IL-17A, IL-17F, IL-21 and IL-22 [55], can promote inflammatory cytokines and chemokines (such as MCP-1 and MIP-2), cyclooxygenase-2, tissue degradation proteases (MMPs) and matrix metalloproteinases, etc., causing inflammatory cell infiltration and tissue destruction [56,57], thus aggravating IBD. Fujino et al. reported that IBD patients had increased numbers of Th17 ​cells compared with healthy controls and active patients had up-regulated levels of Th17 ​cells compared with inactive patients [58]. Treg cells are a type of subgroup of CD4^+^ T cells with immune negative regulation function, secreting anti-inflammatory cytokine IL-10, with the expression of the transcription factor Foxp3. Abnormal function or decrease in Tregs can lead to excessive proliferation of effector cells, causing and aggravating the inflammation of the mucosa. Xu et al. confirmed that Compound Kushen Decoction can significantly improve the symptoms and pathological damage of colitis mice by regulating the balance of Th17/Treg cells in DSS-induced colitis mice, and affecting their immune function [59]. Tao et al. showed that the natural flavonoid glycoside icariin could inhibit the activation of STAT1 and STAT3 and inhibit the response of Th1/Th17 ​cells, thereby reducing experimental colitis in mice [60]. Therefore, the imbalance of Th1/Th2 and Th17/Treg is considered to be an important reason for the progress of UC [61,62]. LUT is a kind of flavonoid existing in numerous Chinese medicinal herbs. Besides its well-known anti-inflammatory activity, LUT has been proved to regulate the CD4^+^ T cell subsets in diseases including acute lung injury, allograft rejection, allergic asthma, etc. [[31], [32], [33]] In our experiment, owing to the UC targeting LUT delivery, we found that the imbalance of Th1/Th2 in spleen and MLNs tended to balance in LUT@TPGS-PBTE NPs treated mice compared to the other groups (Fig. 7A–F). LUT@TPGS-PBTE NPs treated mice showed more obviously down-regulated Th1-related IFN-γ protein and mRNA expressions. Besides, LUT@TPGS-PBTE NPs most effectively inhibited the percentage of Th17 ​cells (Fig. 7G–I) and suppress the levels of IL-17A protein and mRNA secreted by Th17 in the colitis mice. Treg-related cytokine IL-10 and Foxp3 were improved in LUT@TPGS-PBTE NPs treated mice greatly, and the percentage of Tregs in both spleen and MLNs by FCM analysis in LUT@TPGS-PBTE NPs group was significantly elevated and showed no difference from healthy mice (Fig. 7J-L). This shows the recovery function of Tregs. Moreover, compared to LUT ​+ ​TPGS-PBTE NPs, LUT@TPGS-PBTE NPs greatly reduced proinflammatory cytokines (TNF-α, IL-1β, IL-2, IFN-γ and IL-17A) levels and enhanced Th1/Th2/Th17/Treg balance regulation. This is possibly due to the increased LUT accumulation in the inflamed colon as a result of the ROS responsive nature of TPGS-PBTE.

As is known, ulcerative colitis has not only intestinal symptoms, but also some extraintestinal manifestations, such as arthritis, sacroiliitis, decreased hemoglobin, etc. Our study also confirmed the enhancement of systemic inflammation (e.g., WBC, neutrophils, interleukin (IL)-17A, IL-6, interferon-γ, tumor necrosis factor-α in the serum) in DSS-induced mice. The in vivo and ex vivo imaging assay also demonstrated that TPGS-PBTE NPs were more likely to pass through the intestinal blood barrier and reach the peripheral circulation (e.g., liver, spleen and blood), thus LUT@TPGS-PBTE NPs could simultaneously treat colitis and systemic inflammation. Therefore, LUT@TPGS-PBTE NPs exhibited better capability of regulating systemic immunity and increasing hemoglobin levels than that of other groups. Besides, the body weight, DAI score, colon length and pathological score are intuitive indicators of colitis recovery. During the experiments, we observed significant body weight and colon length recovery and decreased DAI score under LUT@TPGS-PBTE NPs treatments (Fig. 5B–E). In addition, H&E of colonic tissues recovered from crypt destruction and reduced lymphocytic infiltration in LUT@TPGS-PBTE NPs groups (Fig. 5F and G).TJ proteins are the major connecting protein between intestinal epithelial cells, represented by claudins, occludin, junctional adhesion molecules, and scaffold protein zonulaoccludins. They are chiefly responsible for regulating paracellular transport and also the main structural component of the formation of barrier function of epithelial cells [39,63]. In our current study, LUT@TPGS-PBTE NPs maintained the intestinal integrity by reducing the loss of claudin-1, occludin and ZO-1, and the tissue injury, diarrhea and bloody stools were significantly relieved. Especially in the LUT@TPGS-PBTE NPs group, TJ proteins loss was the least, showing excellent mucosal protective effect. All of these data indicate that LUT@TPGS-PBTE NPs can serve as a potentially effective agent for the treatment of UC.

## 5 Conclusion

In summary, the present study demonstrated that the ROS responsive system, LUT@TPGS-PBTE NPs, displayed multifaceted protective effects against DSS-induced acute colitis in murine through the regulation of Th1/Th2 and Th17/Treg balance, inhibition of proinflammatory cytokines (TNF-α, IFN-γ, IL-6.etc.), activation of anti-inflammatory factors (IL-10, IL-4), rebuilding of antioxidant/oxidation balance, and enhancement of TJ proteins. Besides, LUT@TPGS-PBTE NPs showed good biosafety, thus they can be a potential strategy for UC therapy. Future studies should focus on the improvement of the encapsulation rate of TPGS-PBTE NPs for luteolin.

## Credit author statement

**Chen Tan:** Conceptualization, Methodology, Software, Writing – original draft. **Heng Fan:** Conceptualization, Data curation, Writing – original draft preparation. **Jiahui Ding,:** Visualization, Investigation, Resources, Software, Validation. **Chaoqun Han,:** Visualization, Investigation, Resources, Software, Validation. **Feng Zhu,:** Visualization, Investigation, Resources, Software, Validation. **Hui Wu,:** Visualization, Investigation, Resources, Software, Validation. **Yujin Liu,:** Visualization, Investigation, Resources, Software, Validation. **Yang Guan** Visualization, Investigation, Resources, Software, Validation. **Wei Zhang:** Visualization, Investigation, Resources, Software, Validation. **Songwei Tan**, Conceptualization, Supervision, Project administration, Funding acquisition, Writing- Reviewing and Editing.**Xiaohua Hou:** Conceptualization, Supervision, Project administration, Funding acquisition, Writing- Reviewing and Editing. **Qing Tang:** Conceptualization, Supervision, Project administration, Funding acquisition, Writing- Reviewing and Editing.

## Declaration of competing interest

The authors declare that they have no known competing financial interests or personal relationships that could have appeared to influence the work reported in this paper.
