# Supplementary material for: ROS-responsive nanoparticles for oral delivery of luteolin and targeted therapy of ulcerative colitis by regulating pathological microenvironment
Source: Mater Today Bio. 2022 Mar 23;14:100246. doi: 10.1016/j.mtbio.2022.100246 (PMC8965165; doi:10.1016/j.mtbio.2022.100246)
Supplement: Multimedia component 1 [file mmc1.docx]

Supplementary data

**ROS-responsive nanoparticles for oral delivery of luteolin and targeted therapy of Ulcerative Colitis by regulating pathological microenvironment**

Chen Tan^2, #^, Heng Fan^1, #^, Jiahui Ding^3^, Chaoqun Han^2^, Yang Guan^4^, Feng Zhu^1^, Hui Wu^1^, Yujin Liu^1^, Wei Zhang ^3^, Xiaohua Hou^2, *^, Songwei Tan ^3, *^, Qing Tang^1, *^

1 Department of Integrated Chinese and Western Medicine, Union Hospital, Tongji Medical College, Huazhong University of Science and Technology, Wuhan 430022, China

2 Department of Gastroenterology, Union Hospital, Tongji Medical College, Huazhong University of Science and Technology, Wuhan 430022, China

3 School of Pharmacy, Tongji Medical College, Huazhong University of Science and Technology, Wuhan 430030, China

4 Academy of Chinese Medical Sciences, Zhejiang Chinese Medical University, Hangzhou 310053, China

*Corresponding author. E-mail addresses: Songwei Tan (tansongwei@gmail.com, tansw@hust.edu.cn), Qing Tang (tqing405@126.com), Xiaohua Hou(houxh@hust.edu.cn).

#These authors have contributed equally to this work

Table S1. List of Primers Used for Quantitative Polymerase Chain Reaction Analysis

| Gene Name |  | Primer sequences (5′ to 3′) |
| --- | --- | --- |
| β-actin | Forward | GTGGGCCGCCCTAGGCACCAG |
|  | Reverse | CTCTTTGATGTCACGCACGATTTC |
| IL-6 | Forward | CTGCAAGAGACTTCCATCCAG |
|  | Reverse | AGTGGTATAGACAGGTCTGTTGG |
| IL-10 | Forward | GGACCAGCTGGACAACATACTGCTA |
|  | Reverse | TCCGATAAGGCTTGGCAACC |
| TNF-α | Forward | CGGGCAGGTCTACTTTGGAG |
|  | Reverse | CAGGTCACTGTCCCAGCATC |
| TGF-β | Forward | TGGAGCAACATGTGGAACTC |
|  | Reverse | CAGCAGCCGGTTACCAAG |
| NF-κB | Forward | ATGGCAGACGATGATCCCTAC |
|  | Reverse | TGTTGACAGTGGTATTTCTGGTG |
| IL-1β | Forward | AAGTATGGGCTGGACTGTTTCTAA |
|  | Reverse | CATGGTTTCTTGTGACCCTGAG |
| Foxp3 | Forward | GTGGGCACGAAGGCAAAG |
|  | Reverse | CCTTGTTTTGCGCTGAGAGTCT |
| IL-17A | Forward | CCTGGCGGCTACAGTGAAG |
|  | Reverse | TTTGGACACGCTGAGCTTTG |
| IL-4 | Forward | GGTCTCAACCCCCAGCTAGT |
|  | Reverse | GCCGATGATCTCTCTCAAGTGAT |
| IFN-γ | Forward | TTGGCTTTGCAGCTCTTCCT |
|  | Reverse | TGACTGTGCCGTGGCAGTA |


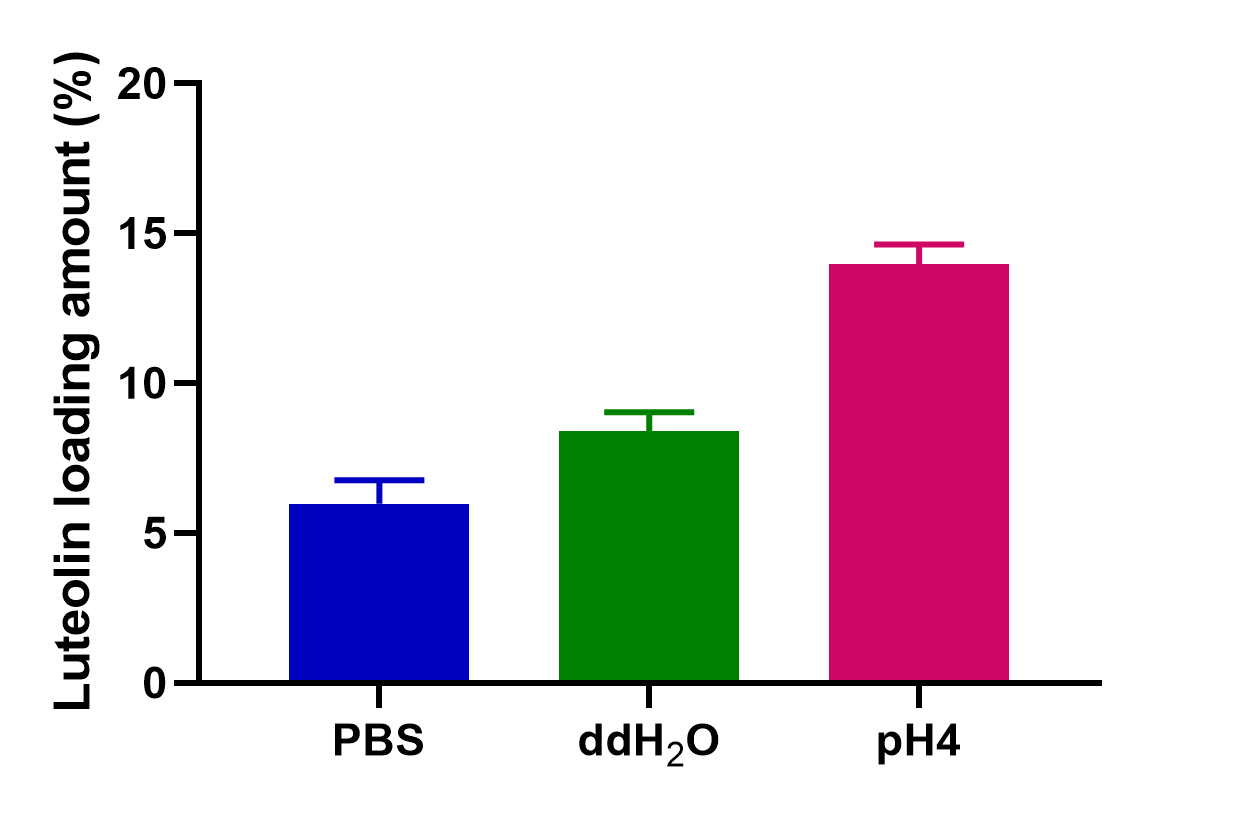


Figure S1. LUT loading amount of LUT@TPGS-PBTE NPs under different preparation condition.


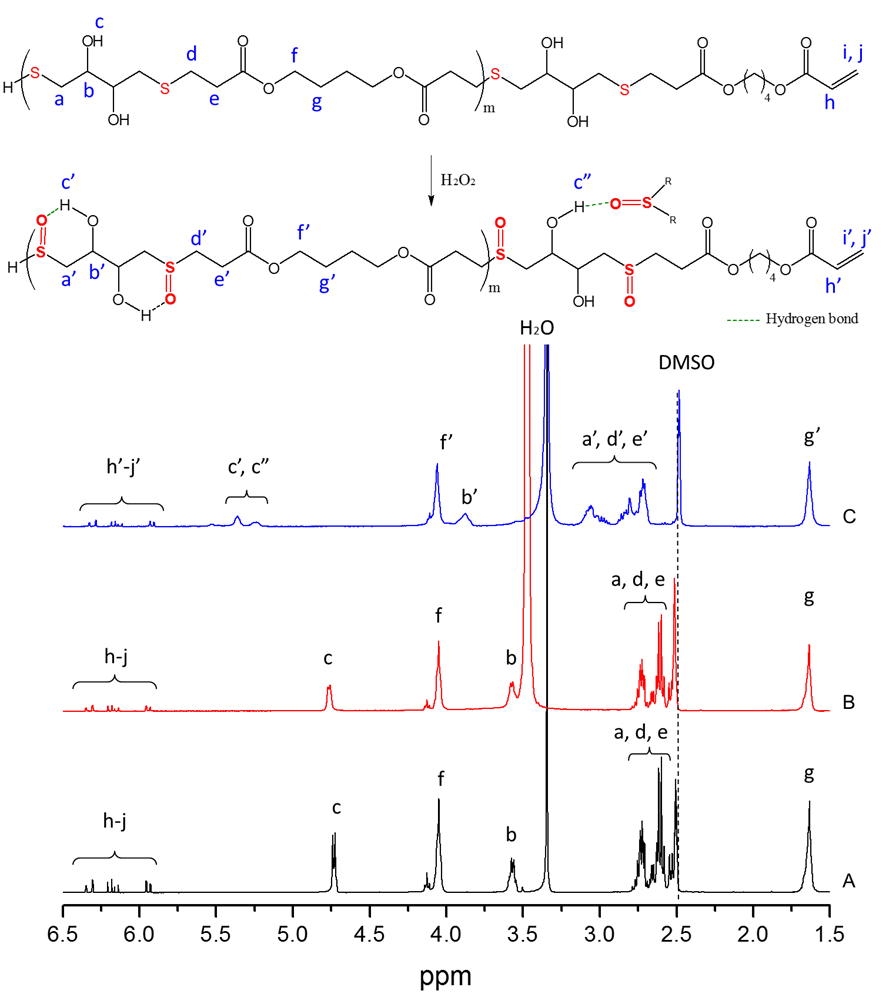


Figure.S2. ROS responsive chemical structure change of PBTE as investigated by ^1^H NMR: A). PBTE, B). PBTE incubated in water for 24 h, C). PBTE incubated in 10mM H_2_O_2_ for 24 h.


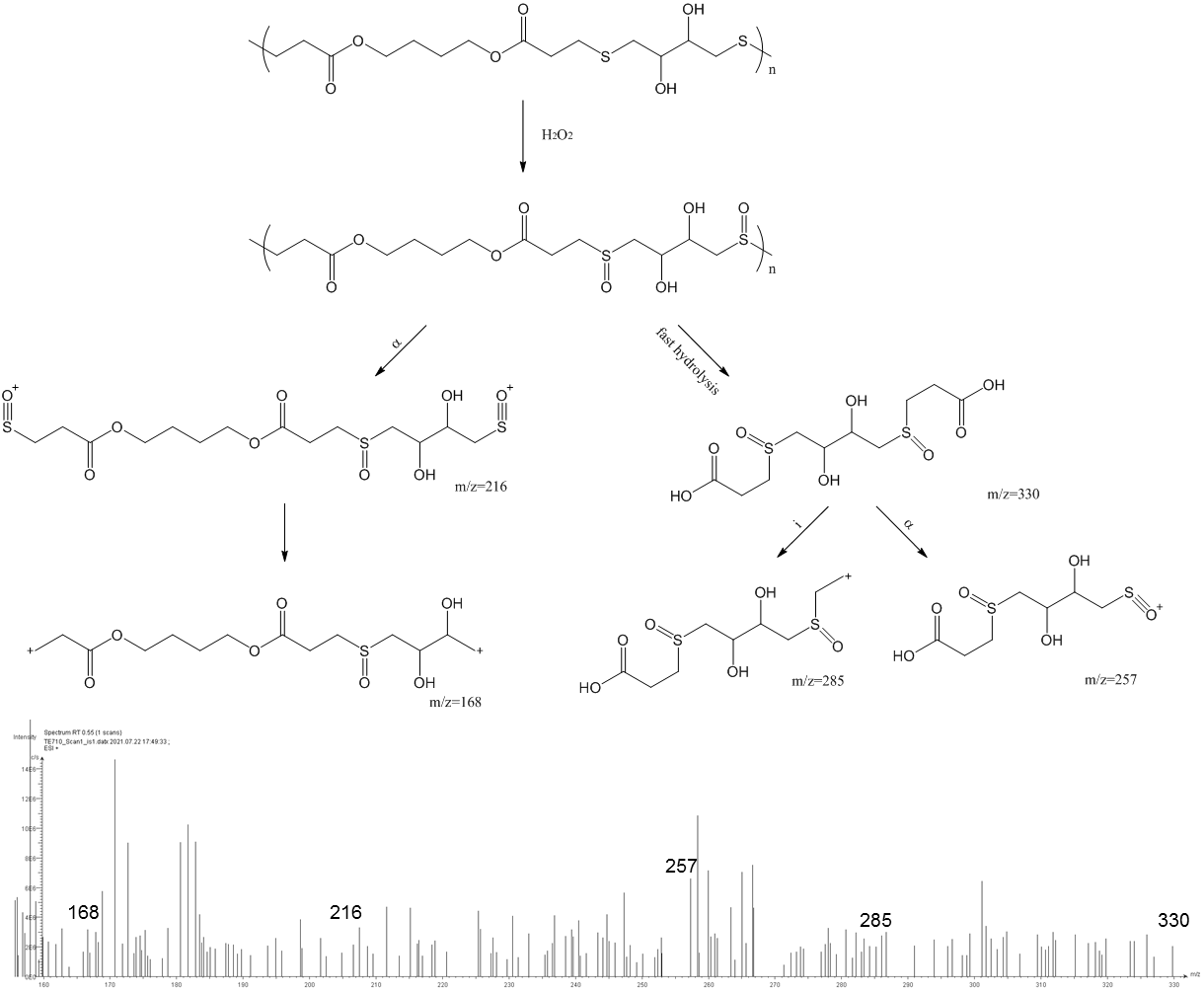


Figure.S3. MS results of PBTE incubated in 10mM H_2_O_2_ for 24h and the possible fragment ion peaks.


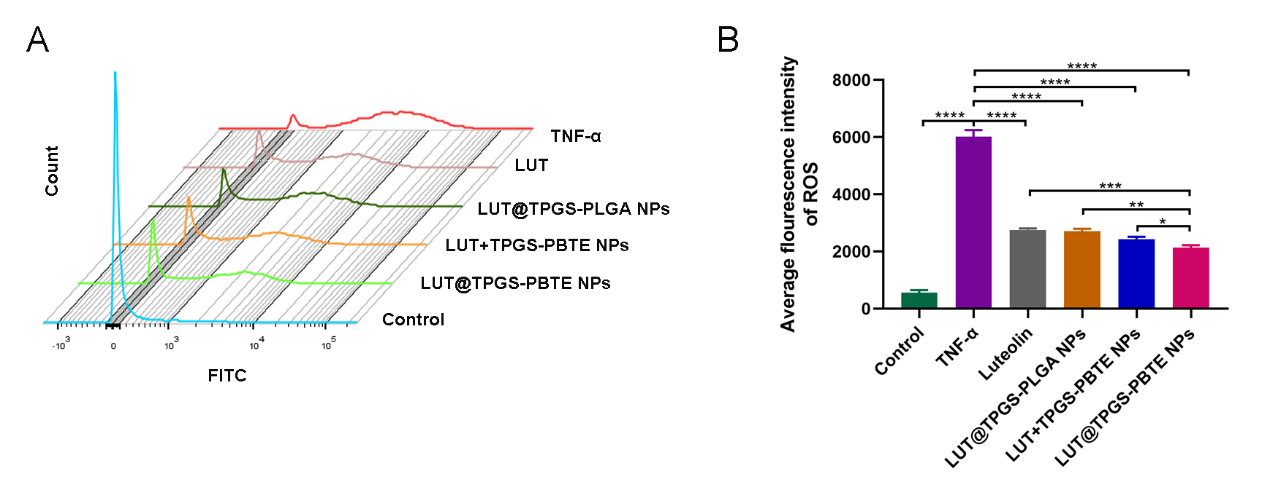


Figure.S4. Reactive oxygen species scavenging effects of LUT@TPGS-PBTE NPs in Caco-2 cells. A) Representative flow cytometric profiles of cellular reactive oxygen species in Caco-2 cells. B) The average fluorescence intensity of ROS.


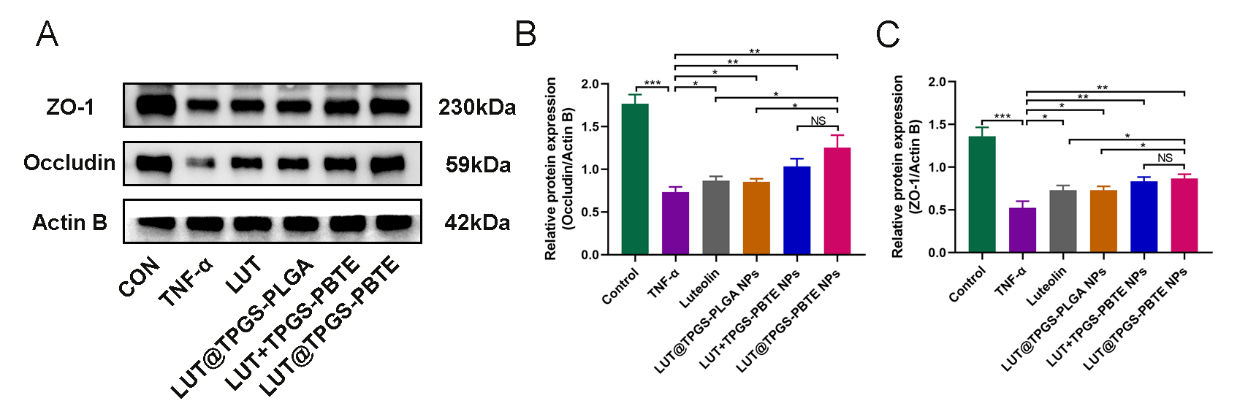


Figure.S5. Intestinal epithelial cell barrier repair effects of LUT@TPGS-PBTE NPs in Caco-2 cells. A) Western blotting analysis of tight junction proteins (occluding and ZO-1). Quantitative analysis of B) occludin and C) ZO-1 protein in Caco-2 cells.
